# Differential age-related transcriptomic analysis of ovarian granulosa cells in Kazakh horses

**DOI:** 10.3389/fendo.2024.1346260

**Published:** 2024-01-30

**Authors:** Wanlu Ren, Jianwen Wang, Yaqi Zeng, Tongliang Wang, Jun Meng, Xinkui Yao

**Affiliations:** ^1^ College of Animal Science, Xinjiang Agricultural University, Urumqi, China; ^2^ Xinjiang Agricultural University, Xinjiang Key Laboratory of Equine Breeding and Exercise Physiology, Urumqi, China

**Keywords:** horse, ovarian granulosa cell, whole transcriptome, ceRNA, PPI

## Abstract

**Introduction:**

The Kazakh horse, renowned for its excellence as a breed, exhibits distinctive reproductive traits characterized by early maturity and seasonal estrus. While normal reproductive function is crucial for ensuring the breeding and expansion of the Kazakh horse population, a noteworthy decline in reproductive capabilities is observed after reaching 14 years of age.

**Methods:**

In this study, ovarian granulosa cells (GCs) were meticulously collected from Kazakh horses aged 1, 2, 7, and above 15 years old (excluding 15 years old) for whole transcriptome sequencing.

**Results:**

The analysis identified and selected differentially expressed mRNAs, lncRNAs, miRNAs, and circRNAs for each age group, followed by a thorough examination through GO enrichment analysis. The study uncovered significant variations in the expression profiles of mRNAs, lncRNAs, miRNAs, and circRNAs within GCs at different stages of maturity. Notably, eca-miR-486-3p and miR-486-y exhibited the highest degree of connectivity. Subsequent GO, KEGG, PPI, and ceRNA network analyses elucidated that the differentially expressed target genes actively participate in signaling pathways associated with cell proliferation, apoptosis, and hormonal regulation. These pathways include but are not limited to the MAPK signaling pathway, Hippo signaling pathway, Wnt signaling pathway, Calcium signaling pathway, Aldosterone synthesis and secretion, Cellular senescence, and NF-kappa B signaling pathway—essentially encompassing signal transduction pathways crucial to reproductive processes.

**Discussion:**

This research significantly contributes to unraveling the molecular mechanisms governing follicular development in Kazakh horses. It establishes and preliminarily validates a differential regulatory network involving lncRNA-miRNA-mRNA, intricately associated with processes such as cell proliferation, differentiation, and apoptosis and integral to the developmental intricacies of stromal follicles. The findings of this study provide a solid theoretical foundation for delving deeper into the realm of reproductive aging in Kazakh mares, presenting itself as a pivotal regulatory pathway in the context of horse ovarian development.

## 1 Introduction

The Kazakh horse, a vital constituent of the equine family, plays an important role in human activities spanning production, daily life, competitive sports, and recreational pursuits. The distinctive seasonal patterns of estrus in Kazakh horses also impact their reproductive capabilities. Throughout the female reproductive cycle, follicles undergo continuous development, initially growing at a similar rate during each estrous period. Follicles, fundamental units of mammalian ovaries, require normal development to produce oocytes capable of fertilization (1). When a follicle, typically the largest, attains a critical size (approximately 35 millimeters in horses), a phenomenon known as diameter deviation occurs. This involves the continued growth of the dominant follicle (occasionally two or three follicles) while all other follicles (subordinate follicles) cease growth and enter a quiescent state (2). GCs, constituting a primary component of the follicle wall (3, 4), play a pivotal role in regulating follicular development. They not only provide nourishment to oocytes but also exert significant control over follicle growth and development through the secretion of hormones, cytokines, and protein regulation. Furthermore, they regulate the meiotic division process of oocytes and orchestrate follicular atresia through apoptosis. Post-maturation, follicular atresia is primarily induced by GCs (5). To discern the potential connection between granulosa cell proliferation and age (6), a growing body of research suggests that follicular atresia is mainly induced by apoptosis in GCs (7, 8). In horses, GCs stand out as the most crucial cell type within follicles, and their quiescence results in a diminishing reproductive function. Studies affirm that follicular atresia predominantly constitutes a hormone-regulated process involving GC apoptosis (9).

In females, oocytes remain in a state of meiotic arrest for several decades, with cycle lengths being similar (22 and 28 days, each featuring a 14-day luteal phase). Only one follicle ovulates per cycle, and the decline in oocyte quality has been identified as a major age-related factor in infertility (10, 11). Aging initiates a cascade of functional declines and diseases, leading to the cessation of reproductive function in both humans and animals. Given the substantial impact of aging on livestock production, the identification of molecular targets associated with aging holds crucial significance. Mares, due to their ovarian dynamics and reproductive capacity resembling humans, serve as crucial animal models for exploring ovarian aging (12). Research on reproductive lifespan remains an underexplored area. Follicles, as functional units of the reproductive lifespan, comprise oocytes (female gametes) and a specialized group of supporting cells. Unraveling the biology of follicles requires more dedicated efforts. In animal models, biological interventions aimed at manipulating pathways related to aging to maintain the quality and quantity of follicles may extend female reproductive and overall health span. Studies indicate intervention strategies targeting the molecular mechanisms driving ovarian aging and menopause (13). Therefore, investigating the mechanisms of follicular development plays a pivotal regulatory role in the reproductive and developmental processes of horses.

In recent years, with the advancements in biotechnology and functional genomics, whole transcriptomics has emerged as a crucial tool for delving into the characteristics and functions of biological cells. By scrutinizing the complete transcriptome of cells, distinctive gene expression patterns specific to each cell type can be discerned, thereby revealing information intricately linked to cell functions. Consequently, the exploration of the whole transcriptome characteristics and functions of ovarian GCs in Kazakh horses holds paramount significance for gaining a profound understanding of the cellular regulatory mechanisms governing the reproductive and developmental processes in this breed. Presently, there is a notable dearth of research in this specific domain. Thus, the primary objective of this study is to investigate the whole transcriptome of ovarian GCs in Kazakh horses, aiming to unravel their transcriptomic characteristics and the functions associated with them. This research endeavors not only to provide crucial support and guidance for the comprehensive study of reproduction and development in equine species but also holds the promise of offering novel insights and methodologies for genetic enhancement and the advancement of reproductive technologies in equine animals.

## 2 Materials and methods

### 2.1 Experimental animals and locations

Experimental Animals: From March 21, 2022, to April 5, 2022, a total of 37 mares were selected from a group of 200 horses. Twenty Kazakh mares were chosen for the study and divided into four groups as follows: Group A - 1 year old (14), five individuals; Group B - 2 years old (15), five individuals; Group C - seven years old (16), five individuals; Group D - above 15 years old (16) (excluding 15 years old), five individuals. The Kazakh horses were sourced from the Tacheng region in Xinjiang, China, and served as the subjects for the research, with experimental samples collected. Throughout the experiment, the mares were housed in individual enclosures in Qiaxia Town, Tacheng City, Xinjiang, China. They were provided with a diet consisting of high-quality dry alfalfa and corn kernels, along with free access to water.

### 2.2 Collection and preservation of ovarian GCs

**Figure 1:**
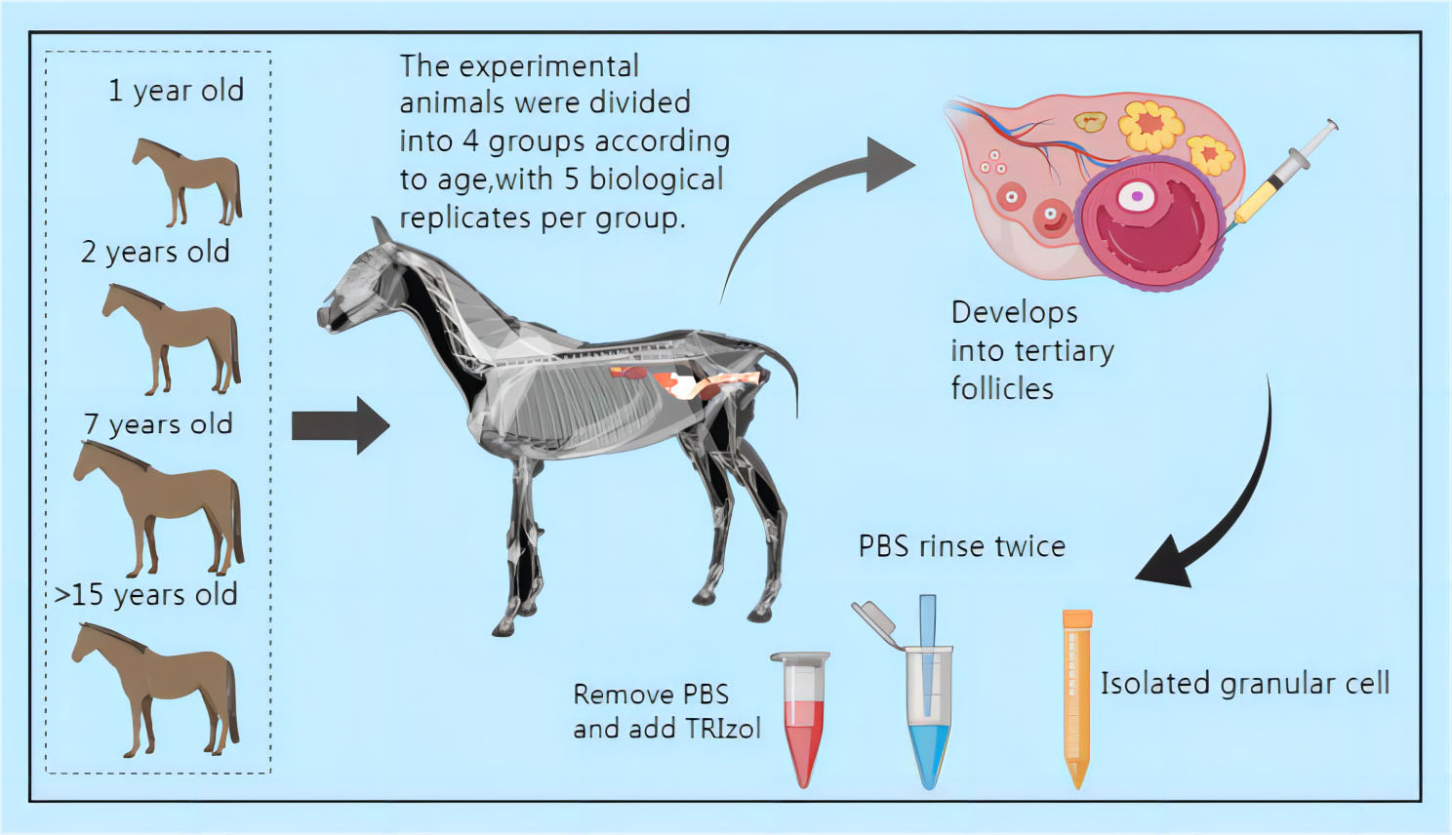
Schematic diagram of sample collection (All mares subjected to surgery for follicular fluid collection received a 14-day intravenous treatment. Daily disinfection and dressing changes were performed at the surgical site. Please refer to the attached document for information on the drugs used.).

From May 8, 2022, to August 8, 2022, ovarian follicles larger than 35mm (Grade III) were identified based on the ovarian follicular development of mares using a B-mode ultrasonic diagnostic instrument. After a 12-hour fasting period, surgical procedures were carried out the next day. The surgery was performed by a licensed veterinarian, assisted by another practicing veterinarian and four assistants. Following the incision of the abdominal wall, follicular fluid was aspirated using a 50 mL syringe and promptly transported to the laboratory for centrifugation. Subsequently, the follicular fluid was discarded, and 2 mL of PBS was added for rinsing before undergoing another round of centrifugation. This process was repeated, and after the final centrifugation, TRIzol was added for preservation. The samples were then stored in liquid nitrogen for future use (See **Figure 1**).

### 2.3 RNA Extraction, library construction, and sequencing

Total RNA was extracted using a Trizol reagent kit (Invitrogen, Carlsbad, CA, USA) following the manufacturer’s protocol. The quality of the RNA was assessed using an Agilent 2100 Bioanalyzer (Agilent Technologies, Palo Alto, CA, USA) and verified through RNase-free agarose gel electrophoresis. Upon RNA extraction, eukaryotic mRNA was enriched using Oligo(dT) beads [For prokaryotes: After total RNA was extracted, prokaryotic mRNA was enriched by removing rRNA by Ribo-ZeroTM Magnetic Kit (Epicentre, Madison, WI, USA)]. Subsequently, the enriched mRNA was fragmented into short fragments using fragmentation buffer and was reverse transcribed into cDNA by using NEBNext Ultra RNA Library Prep Kit for Illumina (NEB #7530, New England Biolabs, Ipswich, MA, USA). The resulting double-stranded cDNA fragments underwent end repair, had an A base added, and were ligated to Illumina sequencing adapters. The ligation reaction was purified with the AMPure XP Beads (1.0X) and subjected to polymerase chain reaction (PCR) for amplification. The resulting cDNA library was sequenced using Illumina Novaseq6000 by Gene Denovo Biotechnology Co. (Guangzhou, China).

### 2.4 Bioinformatics analysis

#### 2.4.1 Filtering of clean reads

Reads obtained from the sequencing machines include raw reads containing adapters or low-quality bases, which can adversely impact subsequent assembly and analysis. To obtain high-quality clean reads, we further filtered the reads using fastp (17) (version 0. 18.0).

#### 2.4.2 Alignment with ribosome RNA

The short-read alignment tool Bowtie2 (18) (version 2.2.8) was employed to map reads to the ribosome RNA (rRNA) database. The reads mapped to rRNA were subsequently removed. The remaining clean reads were further used for assembly and gene abundance calculation.

#### 2.4.3 Alignment with reference genome

An index of the reference genome was constructed, and paired-end clean reads were mapped to the reference genome using HISAT2. 2.4 (19), with other parameters set to default.

#### 2.4.4 Quantification of gene abundance

The mapped reads of each sample were assembled by using StringTie v1.3. 1 (20, 21) in a reference-based approach. For each transcription region, an FPKM (fragment per kilobase of transcript per million mapped reads) value was calculated to quantify its expression abundance and variations using RSEM (22) software.

The FPKM formula is shown as follows:


$$\text{FPKM}=\frac{10^{6}\text{C}}{\text{NL}/10^{3}}$$

Where FPKM(A) represents the expression of gene A, C represents the number of fragments mapped to gene A, N represents the total number of fragments mapped to reference genes, and L represents the number of bases on gene A. The FPKM method effectively eliminates the influence of different gene lengths and sequencing data amounts on the calculation of gene expression. Consequently, the calculated gene expression can be directly used to compare differences in gene expression among samples.

### 2.5 Relationship analysis of samples

#### 2.5.1 Correlation analysis of replicas

Correlation analysis was performed using R to assess the reliability and operational stability of two parallel experiments. The correlation coefficient between replica samples was calculated to evaluate repeatability. A correlation coefficient approaching 1 indicates higher repeatability between the two parallel experiments.

#### 2.5.2 Principal component analysis

Principal component analysis (PCA) was performed in this study using the R package gmodels (http://www.rproject.org/). PCA is a statistical procedure that converts hundreds of thousands of correlated variables (gene expression) into a set of values of linearly uncorrelated variables known as principal components. PCA is widely employed to elucidate the structure/relationship among the samples/data.

### 2.6 Differentially expressed genes

Differential expression analysis of RNAs was performed using DESeq2 (23) software between different groups (and by edgeR (24) between two samples). Genes/transcripts with a false discover rate FDR parameter below 0.05 and an absolute fold change≥2 were considered differentially expressed genes/transcripts.

#### 2.6.1 GO enrichment analysis

Firstly, all DEGs were mapped to GO terms in the Gene Ontology database (http://www.geneontology.org/), and gene numbers were calculated for every term. Significantly enriched GO terms in DEGs, compared to the genome background, were determined using the hypergeometric test (25). The calculating formula for the P-value is:


$$P=1-\sum_{i=0}^{m-1}\frac{\left(\begin{array}{c} M\\ i \end{array}\right)\left(\begin{array}{c} N-M\\ n-i \end{array}\right)}{\left(\begin{array}{c} N\\ n \end{array}\right)}$$

Where N represents the total number of genes with GO annotation; n represents the number of DEGs in N; M represents the total number of genes annotated to certain GO terms; and m represents the number of DEGs in M. The calculated p-value underwent False Discovery Rate (FDR) correction, with an FDR ≤ 0.05 threshold. GO terms meeting this condition were defined as significantly enriched GO terms in DEGs. This analysis helped identify the primary biological functions exercised by DEGs.

#### 2.6.2 Pathway enrichment analysis

KEGG, a prominent public pathway-related database, was employed for pathway enrichment analysis, identifying significantly enriched metabolic or signal transduction pathways in DEGs compared with the whole genome background (26). The calculating formula is the same as that in GO analysis.


$$P=1-\sum_{i=0}^{m-1}\frac{\left(\begin{array}{c} M\\ i \end{array}\right)\left(\begin{array}{c} N-M\\ n-i \end{array}\right)}{\left(\begin{array}{c} N\\ n \end{array}\right)}$$

Where N represents the total number of genes with KEGG annotation, n represents the number of DEGs in N, M represents the number of all genes annotated to specific pathways, and m represents the number of DEGs in M. The calculated p-value underwent FDR correction, with an FDR ≤ 0.05 threshold. Pathways meeting this condition were defined as significantly enriched pathways in DEGs.

### 2.7 Protein-protein interaction

The Protein-Protein Interaction network was constructed using String v10 (27), designating genes as nodes and interaction as lines. The resulting network file was visualized using Cytoscape (v3.7.1) (28) software to depict core and hub gene biological interactions.

### 2.8 Single-nucleotide polymorphism analysis

The GATK (29) (version 3.4-46) was used for variant calling of transcripts, and ANNOVAR was used for SNP/InDel annotation. The function, genome site, and type of variation of SNPs were also analyzed.

### 2.9 Validation through RT-qPCR

To confirm the accuracy of the sequencing data, we randomly chose a set of six differentially expressed mRNAs for fluorescent quantitative testing using RT-qPCR. The reference genes employed for this validation were *GADPH* and *β-Actin*. For detailed primer sequences, please refer to **Supplementary Materials** (see **Supplementary Table S1** for information on differentially expressed mRNA primers, materials and methods).

## 3 Results

### 3.1 Comparison of clean reads to the reference genome

This study reveals that, through transcriptome sequencing of GCs from 20 Kazakh mares, 50.73% to 92.82% of clean reads were successfully aligned to the reference genome (Ensembl_release100). Based on the alignment of transcriptome data with the reference genome, a total of 291 new genes were annotated, and the SNP count ranged from 1,053,699 to 2,281,283 (please refer to **Supplementary Table S2**).

### 3.2 Identification and analysis of differentially expressed mRNAs

**Figure 2:**
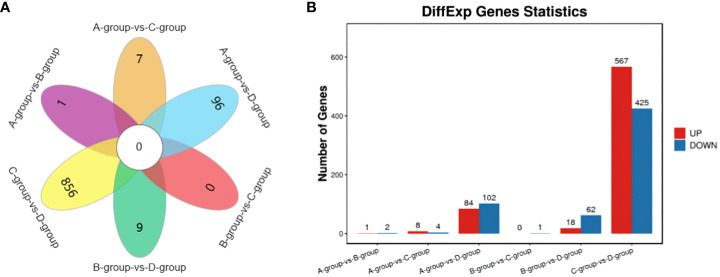
Statistics of differentially expressed mRNAs in each comparison group. **(A)** mRNAs Venn diagram of differences between groups; **(B)** mRNAs statistical histogram of differences among groups.

In the Group A vs Group B comparison, three mRNAs were identified (one upregulated and two downregulated): *SNRPN*, *RPS17*, and *LRP2*. For the Group A vs Group C comparison, twelve mRNAs were identified (eight upregulated and four downregulated), including *NID1*, *SNRPN*, and *COL1A1*, among others. In the Group A vs Group D comparison, a total of 186 mRNAs were identified (84 upregulated and 102 downregulated), such as *LARGE2*, *CDH24*, *TENM4*, and others. In the Group B vs Group C comparison, one mRNA was identified (no upregulation and one downregulation), specifically *MMP12*. For the Group B vs Group D comparison, a total of 80 mRNAs were identified (18 upregulated and 62 downregulated), including *IHH*, *TENM4*, and *CDH24*, among others. Lastly, in the Group C vs Group D comparison, 992 mRNAs were identified (567 upregulated and 425 downregulated), such as *TENM4*, *SLC39A10*, *CTNNAL1*, and others. Group C vs Group D exhibited the highest number of differentially expressed mRNAs, as depicted in **Figure 2**.

### 3.3 Identification and classification of lncRNA

**Figure 3:**
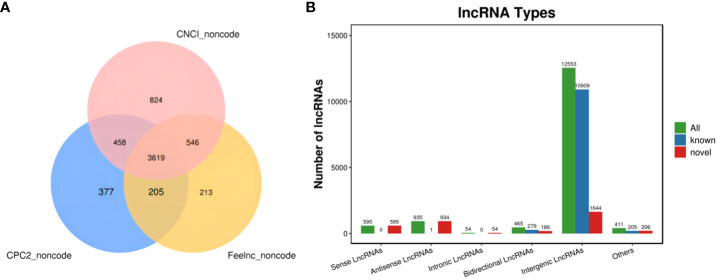
LncRNA identification and analysis. **(A)** Venn diagram illustrating lncRNA coding potential; **(B)** Bar Chart displaying lncRNA types.

The new LncRNAs have been categorized into five major classes based on their relative positions in the genome compared to protein-coding genes: Intergenic Long Non-Coding RNAs (Intergenic LncRNAs): 12,553; Bidirectional Long Non-Coding RNAs (Bidirectional LncRNAs): 465; Intronic Long Non-Coding RNAs (Intronic LncRNAs): 54; Antisense Long Non-Coding RNAs (Antisense LncRNAs): 935; Sense Overlapping Long Non-Coding RNAs (Sense Overlapping LncRNAs): 595. Transcript reconstruction using stringtie and prediction of transcript coding potential through CPC2, CNCI, and Feelnc software resulted in reliable predictions, as depicted in **Figure 3**.

**Figure 4:**
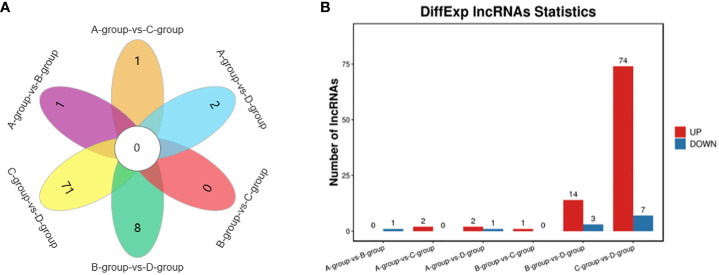
Statistics of differentially expressed LncRNAs in each comparison group. **(A)** LncRNAs Venn diagram of differences between groups; **(B)** LncRNAs statistical histogram of differences among groups.

In Group A vs Group B, one lncRNA (MSTRG.14266.1) was identified, showing no upregulation and one downregulation. In Group A vs Group C, two lncRNAs (ENSECAT00000071442 and MSTRG.4012.8) were identified, both exhibiting upregulation and no downregulation. In Group A vs Group D, three lncRNAs (MSTRG.40650.1, ENSECAT00000049786, MSTRG.49929.4) were identified, with two upregulated and one downregulated. In Group B vs Group C, one lncRNA (ENSECAT00000071442) was identified, showing upregulation and no downregulation. Regarding Group B vs Group D, a total of 17 lncRNAs (e.g., MSTRG.24142.1, MSTRG.44109.1, MSTRG.14314.1) were identified, with 14 upregulated and three downregulated. In Group C vs Group D, a total of 81 lncRNAs (e.g., MSTRG.3596.12, MSTRG.39723.1, MSTRG.10102.1) were identified, with 74 upregulated and seven downregulated. The comparison of Group C vs Group D exhibited the highest number of differentially expressed lncRNAs, as depicted in **Figure 4**.

### 3.4 Identification and analysis of differentially expressed miRNAs

**Figure 5:**
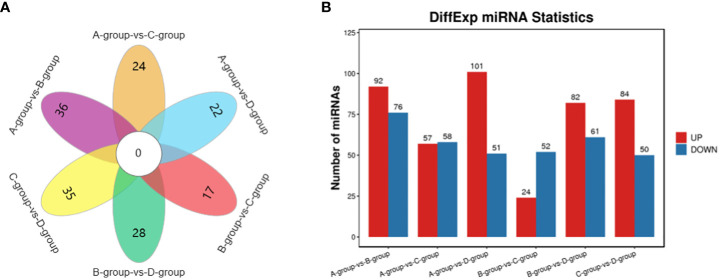
Statistics of differentially expressed miRNAs in each comparison group. **(A)** miRNAs Venn diagram of differences between groups; **(B)** miRNAs statistical histogram of differences among groups.

In the Group A vs B group comparison, a total of 168 miRNAs were identified (92 upregulated and 76 downregulated), including MSTRG.10102.1, eca-miR-146a, eca-miR-143, and others. For Group A vs Group C, 115 miRNAs were identified (57 upregulated and 58 downregulated), such as eca-miR-146a, novel-m0007-3p, eca-miR-450b-5p, and others. In Group A vs Group D, 152 miRNAs were identified (101 upregulated and 51 downregulated), including eca-miR-199a-5p, eca-miR-143, eca-miR-199a-3p, and others. In Group B vs Group C, 76 miRNAs were identified (24 upregulated and 52 downregulated), including eca-miR-451, eca-miR-199a-3p, eca-miR-199b-3p, and others. In Group B vs Group D, a total of 143 miRNAs were identified (82 upregulated and 61 downregulated), such as miR-99-z, eca-miR-142-5p, miR-486-x, and others. Lastly, in Group C vs Group D, 134 miRNAs were identified (84 upregulated and 50 downregulated), including eca-miR-199a-5p, eca-miR-20a, and miR-2478-z, as depicted in **Figure 5**.

### 3.5 Identification and analysis of differentially expressed circRNAs

**Figure 6:**
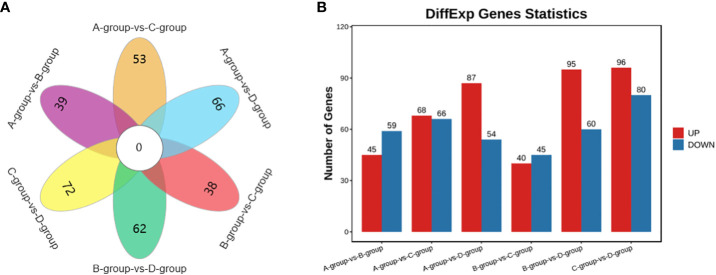
Statistics of differentially expressed circRNAs in each comparison group. **(A)** circRNAs Venn diagram of differences between groups; **(B)** circRNAs statistical histogram of differences among groups.

In the Group A vs Group B comparison, a total of 104 circRNAs were identified (45 upregulated and 59 downregulated), including *TEX14*, *PCMT1*, *SLAMF6*, and others. For Group A vs Group C, 134 circRNAs were identified (68 upregulated and 66 downregulated), such as *MERTK*, *EIF4B*, *PSD3*, and others. In Group A vs Group D, 141 circRNAs were identified (87 upregulated and 54 downregulated), including *SEC24B*, *ACTB*, *THBS1*, and others. Regarding the Group B vs Group C comparison, 85 circRNAs were identified (40 upregulated and 45 downregulated), including *PPM1D*, *MYO6*, *USP7*, and others. In Group B vs Group D, a total of 155 circRNAs were identified (95 upregulated and 60 downregulated), including *CPSF6*, *MDN1*, *GNS*, and others. Lastly, in Group C vs Group D, 176 circRNAs were identified (96 upregulated and 80 downregulated), featuring *VCAN*, *MYO1D*, and *STAG1*, as depicted in **Figure 6**.

**Figure 7:**
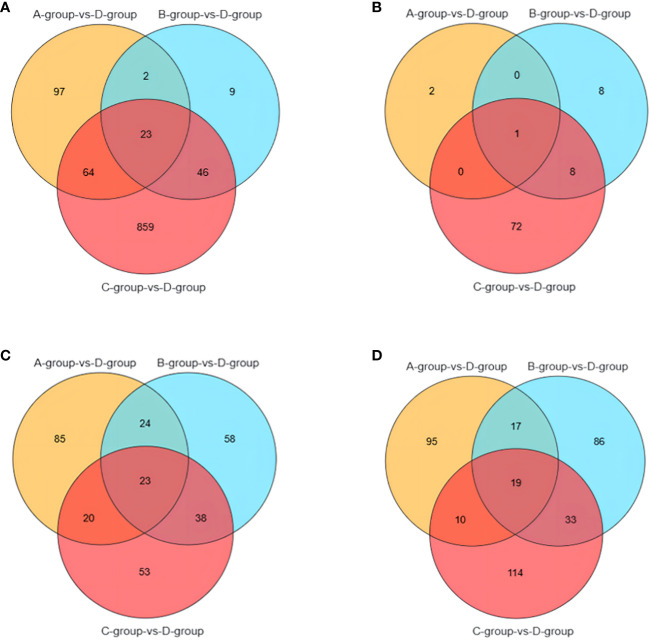
Venn diagrams of: **(A)** the shared differentially expressed mRNAs in Group A vs Group D, Group B vs Group D, and Group C vs Group D; **(B)** the shared differentially expressed lncRNAs in Group A vs Group D, Group B vs Group D, and Group C vs Group D; **(C)** the shared differentially expressed miRNAs in Group A vs Group D, Group B vs Group D, and Group C vs Group D; **(D)** the shared differentially expressed circRNAs in Group A vs Group D, Group B vs Group D, and Group C vs Group D.

It is noteworthy that there are 23 differential genes for mRNA in Group A vs Group D, Group B vs Group D, and Group C vs Group D, respectively. For lncRNA, there is one differential gene in each of the Group A vs Group D, Group B vs Group D, and Group C vs Group D comparisons. Additionally, miRNA exhibits 23 differential genes in Group A vs Group D, Group B vs Group D, and Group C vs Group D, respectively. As for circRNA, there are 19 differential genes in Group A vs Group D, Group B vs Group D, and Group C vs Group D, respectively. These shared differentially expressed mRNA, lncRNA, miRNA, and circRNA can be further investigated as key regulators in the aging process of mare reproduction, influencing age-related changes in ovarian function and follicular development, as illustrated in **Figure 7**.

### 3.6 Enrichment analysis of differentially expressed mRNAs

**Figure 8:**
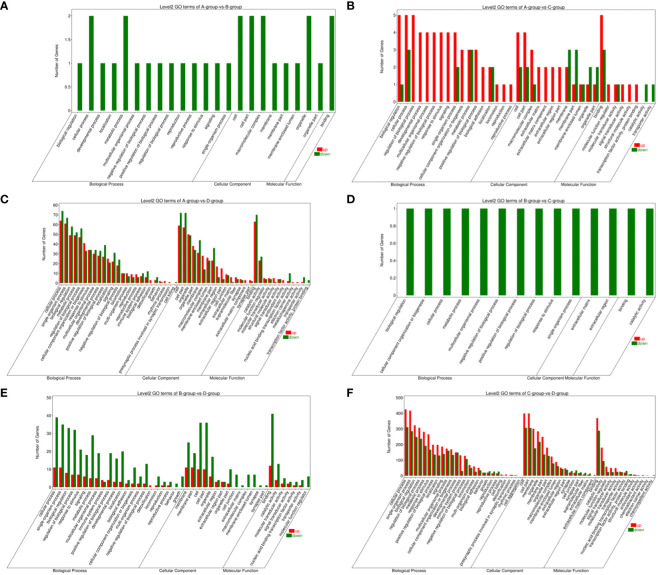
GO enrichment analysis of differentially expressed mRNAs. **(A)** Group A vs Group B; **(B)** Group A vs Group C; **(C)** Group A vs Group D; **(D)** Group B vs Group C; **(E)** Group B vs Group D; **(F)** Group C vs Group D.

In the Group A vs Group B, Group A vs Group C, Group A vs Group D, Group B vs Group C, Group B vs Group D, and Group C vs Group D comparisons, 2, 9, 146, 1, 55, and 808 differentially expressed mRNAs were respectively annotated in the GO database. In Group A vs Group B, significant enrichments were observed in U2-type prespliceosome (Cellular Component - CC), U5 snRNP (CC), chaperone binding (Molecular Function - MF), positive regulation of oligodendrocyte progenitor proliferation (Biological Process - BP), and regulation of oligodendrocyte progenitor proliferation (BP). In Group A vs Group C, major enrichments were identified in proteinaceous extracellular matrix (CC), Wnt-protein binding (MF), acetyltransferase activator activity (MF), laminin-1 binding (MF), and Wnt signaling pathway (BP). In Group A vs Group D, enrichments were prominent in cell junction (CC), germinal vesicle (CC), protein homodimerization activity (MF), protein binding (MF), cellular component organization (BP), and cellular component organization or biogenesis (BP). In Group B vs Group C, notable enrichments were found in extracellular matrix (CC), metalloendopeptidase activity (MF), negative regulation of hydrogen peroxide metabolic process (BP), and regulation of hydrogen peroxide metabolic process (BP). In Group B vs Group D, major enrichments included cell surface (CC), cell junction (CC), calcium ion binding (MF), vascular endothelial growth factor receptor 2 binding (MF), and cell adhesion molecule binding (MF). In Group C vs Group D, enrichments were predominantly observed in cell surface (CC), membrane (CC), endomembrane system (CC), protein binding (MF), binding (MF), cytoskeletal protein binding (MF), positive regulation of response to stimulus (BP), and regulation of response to stimulus (BP) (see **Figure 8**).

Bar chart of GO enrichment classification: (The horizontal axis represents secondary GO terms, and the vertical axis represents the number of differentially expressed genes in that term, with red indicating upregulation and green indicating downregulation; the same applies below).

**Figure 9:**
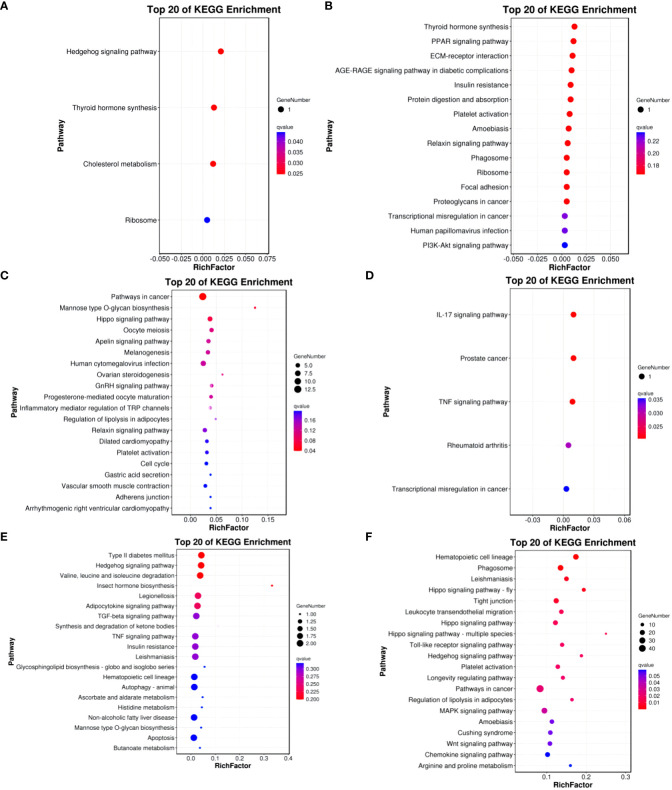
KEGG enrichment analysis of differentially expressed mRNAs. **(A)** Group A vs Group B; **(B)** Group A vs Group C; **(C)** Group A vs Group D; **(D)** Group B vs Group C; **(E)** Group B vs Group D; **(F)** Group C vs Group D.

In the Group A vs Group B, Group A vs Group C, Group A vs Group D, Group B vs Group C, Group B vs Group D, and Group C vs Group D comparisons, differentially expressed mRNAs were significantly enriched in 4, 0, 1, 5, 0, and 15 signaling pathways, respectively (q-value < 0.05). In Group A vs Group B, the differentially enriched pathways include Hedgehog signaling pathway, Thyroid hormone synthesis, Cholesterol metabolism, and Ribosome. In Group A vs Group D, the differentially enriched pathway is Pathways in cancer. In Group B vs Group C, differentially enriched pathways include the IL-17 signaling pathway, Prostate cancer, TNF signaling pathway, Rheumatoid arthritis, and Transcriptional misregulation in cancer. In Group C vs Group D, differentially enriched pathways include Hematopoietic cell lineage, Phagosome, Leishmaniasis, Hippo signaling pathway - fly, Tight junction, Leukocyte transendothelial migration, Hippo signaling pathway, Hippo signaling pathway - multiple species, Toll-like receptor signaling pathway, Hedgehog signaling pathway, Platelet activation, Longevity regulating pathway, Pathways in cancer, Regulation of lipolysis in adipocytes, and MAPK signaling pathway (refer to **Figure 9**).

KEGG enrichment bubble chart: (Constructed using the 20 pathways with the lowest Q-values for visualization. The vertical axis represents pathways, the horizontal axis represents the enrichment factor (the ratio of differentially expressed genes in that pathway to the total number in that pathway), the bubble size indicates quantity, and the color becomes deeper red as the Q-value decreases; the same principles apply below).

### 3.7 Enrichment analysis of differentially expressed lncRNA target genes

Predictions for target genes of differentially expressed lncRNAs between groups were made. Subsequently, GO functional and KEGG pathway enrichment analyses were performed on these target genes to delve into the roles of the identified differentially expressed lncRNAs during the ovarian follicular development of Kazakh horses at various ages. Cis-target gene predictions were based on the positional relationship between lncRNAs and mRNAs, considering mRNAs detected within a specified range upstream and downstream of lncRNAs as their target genes. Results showed that within the 10 kb upstream and downstream range of lncRNAs, a total of 3067 lncRNAs corresponded to 3006 protein-coding genes, forming 3972 lncRNA-mRNA pairs. Additionally, trans-expression target gene prediction, based on the correlation between lncRNAs and mRNAs (with a screening criterion of an absolute value of Pearson correlation coefficient greater than 0.95), identified 1235 potential regulatory relationships between 50 lncRNAs and 252 mRNA targets.

**Figure 10:**
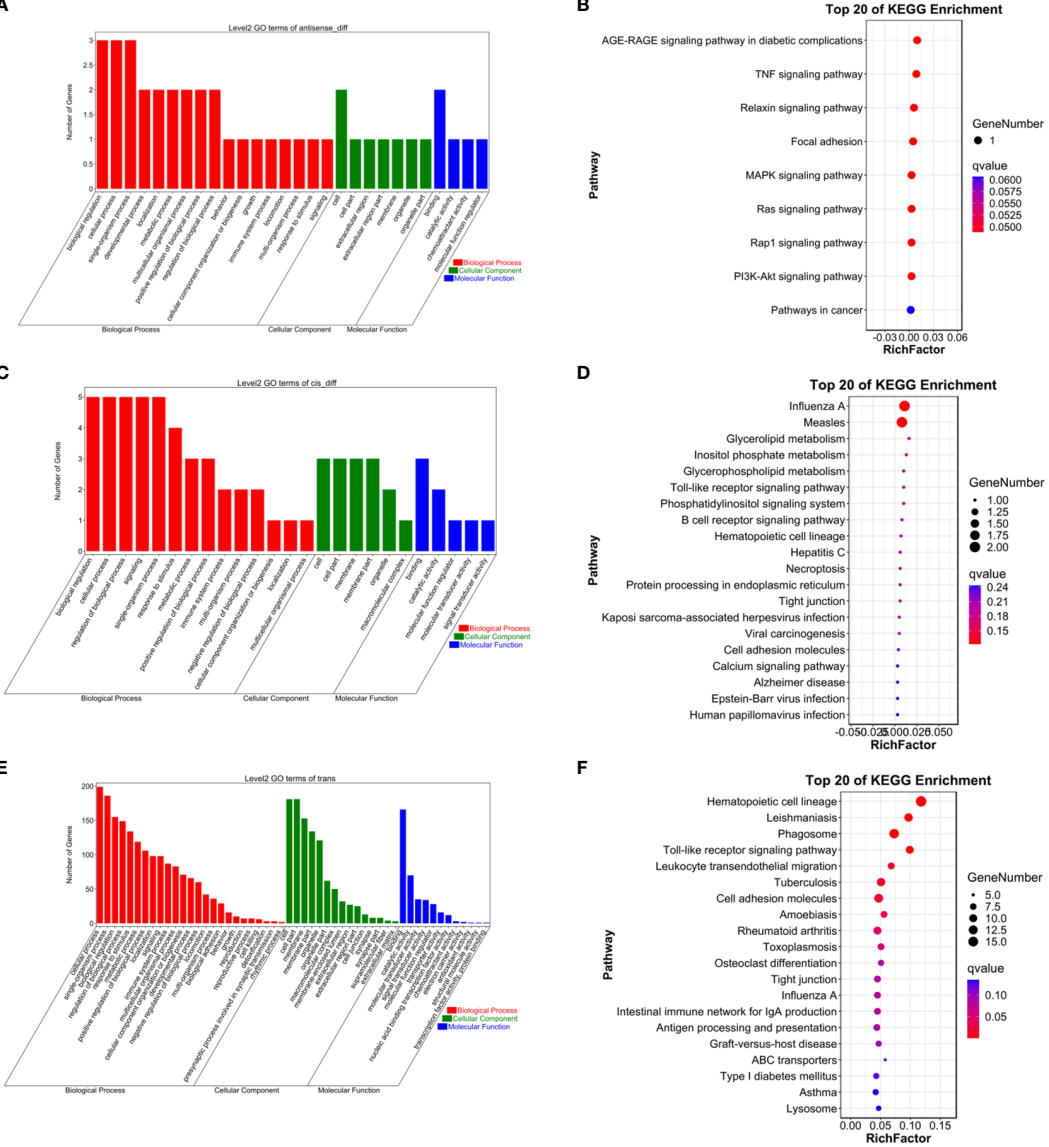
**(A)** Antisense GO enrichment analysis of differentially expressed lncRNAs target gene. **(B)** Antisense KEGG enrichment analysis of differentially expressed lncRNAs target gene. **(C)** CIS GO enrichment analysis of differentially expressed lncRNAs target gene. **(D)** CIS KEGG enrichment analysis of differentially expressed lncRNAs target gene. **(E)** Trans GO enrichment analysis of differentially expressed lncRNAs target gene **(F)** Trans KEGG enrichment analysis of differentially expressed lncRNAs target gene.

To gain a more comprehensive understanding of the functional roles of these differentially expressed lncRNAs, GO functional enrichment and KEGG pathway analyses were separately conducted for cis and trans target genes. The results revealed that under the cis-regulatory mode, the target genes of differentially expressed lncRNAs were significantly enriched in 138 GO terms (P<0.05, **Figures 2**–**9**). These terms included 123 BP, 9 MF, and 6 CC. Notably, some of the top-ranking GO terms included “positive regulation of interferon-alpha biosynthetic process” (BP), “negative regulation of B cell receptor signaling pathway” (BP), “cellular response to external stimulus” (BP), “regulation of interferon-alpha biosynthetic process” (BP), “double-stranded RNA binding” (MF), “eukaryotic translation initiation factor 2alpha kinase activity” (MF), “siRNA binding” (MF), “pattern recognition receptor activity” (MF), “kinase activity” (MF), “early phagosome” (CC), “endosome” (CC), “phagocytic vesicle” (CC), “recycling endosome” (CC). In addition, a total of KEGG pathways showed significant enrichment (P<0.05, **Figure 10**). Among the top-ranking KEGG pathways were “Influenza A,” “Measles,” and “Glycerolipid metabolism” signaling pathways. Notably, enrichment was also observed in some common signaling pathways associated with testicular tissue development or spermatogenesis, such as “Cell adhesion molecules” and “Calcium signaling pathway” (see **Figure 10**).

Under the trans-regulatory mode, the target genes of differentially expressed lncRNAs were notably enriched in 138 GO terms (*P*<0.05, **Figure 10**). These included 123 BP, 9 MF, and 6 CC. Important GO terms consisted of “positive regulation of interferon-alpha biosynthetic process” (BP), “negative regulation of B cell receptor signaling pathway” (BP), “cellular response to external stimulus” (BP), “interferon-alpha biosynthetic process” (BP), “regulation of interferon-alpha biosynthetic process” (BP), “double-stranded RNA binding” (MF), “eukaryotic translation initiation factor 2alpha kinase activity” (MF), “siRNA binding” (MF), “pattern recognition receptor activity” (MF), “transferase activity, transferring phosphorus-containing groups” (MF), “early phagosome” (CC), “endosome” (CC), “phagocytic vesicle” (CC), “recycling endosome” (CC), “endocytic vesicle” (CC), and others. Moreover, a total of 23 KEGG signaling pathways were identified, including “Influenza A,” “Measles,” and “Glycerolipid metabolism,” among others (refer to **Figure 10**).

### 3.8 Enrichment analysis of differentially expressed miRNA target genes

**Figure 11:**
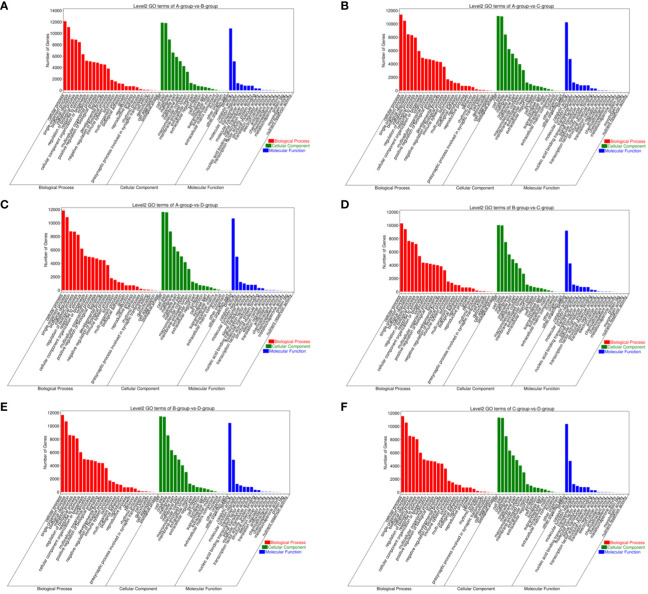
GO enrichment analysis of differentially expressed miRNAs target gene. **(A)** Group A vs Group B; **(B)** Group A vs Group C; **(C)** Group A vs Group D; **(D)** Group B vs Group C; **(E)** Group B vs Group D; **(F)** Group C vs Group D.

For Group A vs Group B, Group A vs Group C, Group A vs Group D, Group B vs Group C, Group B vs Group D, and Group C vs Group D, there were 13,363, 12,551, 13,056, 11,287, 12,854, and 12,730 annotated differentially expressed miRNA target genes in the GO database, respectively. In Group A vs Group B, differentially expressed miRNA target genes were enriched in “cell cortex” (CC), “cytoplasm” (CC), “endomembrane system” (CC), “binding” (MF), “protein binding” (MF), “carbohydrate derivative binding” (MF), “single-multicellular organism process” (BP), “regulation of biological quality” (BP), “enzyme-linked receptor protein signaling pathway” (BP). In Group A vs Group C, differentially expressed miRNA target genes were enriched in “binding” (MF), “protein binding” (MF), “carbohydrate derivative binding” (MF), “single-multicellular organism process” (BP), “regulation of biological quality” (BP), “enzyme-linked receptor protein signaling pathway” (BP). In Group A vs Group D, differentially expressed miRNA target genes were enriched in “cytoplasm” (CC), “endomembrane system” (CC), “cell cortex” (CC), “binding” (MF), “protein binding” (MF), “Ras GTPase binding” (MF), “single-multicellular organism process” (BP), “regulation of biological quality” (BP), “cellular response to organic substance” (BP). In Group B vs Group C, differentially expressed miRNA target genes were enriched in “intracellular” (CC), “intracellular part” (CC), “cytoplasm” (CC), “binding” (MF), “protein binding” (MF), “growth factor binding” (MF), “single-multicellular organism process” (BP), “enzyme-linked receptor protein signaling pathway” (BP), “regulation of biological quality” (BP). In Group B vs Group D, differentially expressed miRNA target genes were enriched in “cell cortex” (CC), “focal adhesion” (CC), “cytoplasm” (CC), “protein binding” (MF), “binding” (MF), “Ras GTPase binding” (MF), “single-multicellular organism process” (BP), “system development” (BP), “multicellular organism development” (BP). In Group C vs Group D, differentially expressed miRNA target genes were enriched in “cell cortex” (CC), “focal adhesion” (CC), “cytoplasm” (CC), “protein binding” (MF), “binding” (MF), “Ras GTPase binding” (MF), “single-multicellular organism process” (BP), “anatomical structure development” (BP), “system development” (BP) (refer to **Figure 11**).

**Figure 12:**
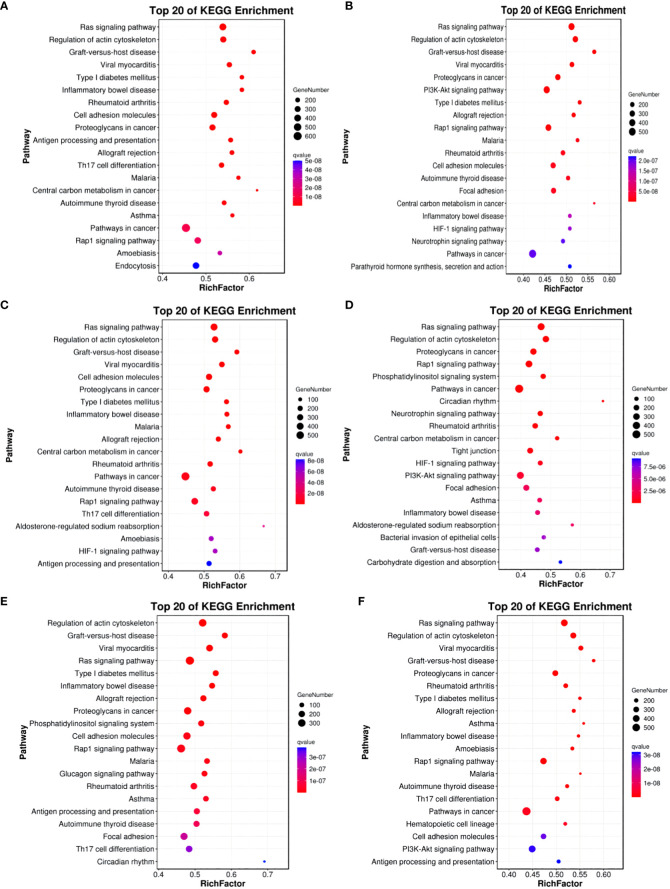
KEGG enrichment analysis of differentially expressed miRNAs target gene. **(A)** Group A vs Group B; **(B)** Group A vs Group C; **(C)** Group A vs Group D; **(D)** Group B vs Group C; **(E)** Group B vs Group D; **(F)** Group C vs Group D.

In comparisons between Group A vs Group B, Group A vs Group C, Group A vs Group D, Group B vs Group C, Group B vs Group D, and Group C vs Group D, the target genes of differentially expressed miRNAs are significantly enriched in 137, 135, 140, 118, 113, and 113 pathways, respectively (q-value < 0.05). Notably, the enriched pathways vary among the comparisons: In Group A vs Group B, enriched pathways include the Ras signaling pathway, Regulation of actin cytoskeleton, and Graft-versus-host disease. In Group A vs Group C, enriched pathways involve the Ras signaling pathway, Regulation of actin cytoskeleton, and Graft-versus-host disease. In Group A vs Group D, enriched pathways encompass the Ras signaling pathway, Regulation of actin cytoskeleton, and Graft-versus-host disease. In Group B vs Group C, enriched pathways consist of the Ras signaling pathway, Regulation of actin cytoskeleton, and Proteoglycans in cancer. In Group B vs Group D, enriched pathways include Regulation of actin cytoskeleton, Graft-versus-host disease, Viral myocarditis, and ECM-receptor interaction. In Group C vs Group D, enriched pathways involve the Ras signaling pathway, Regulation of actin cytoskeleton, Cell adhesion molecules, PI3K-Akt signaling pathway, Calcium signaling pathway, AMPK signaling pathway, MAPK signaling pathway, and GnRH signaling pathway. (refer to **Figure 12**).

### 3.9 Enrichment analysis of differentially expressed circRNA target genes

**Figure 13:**
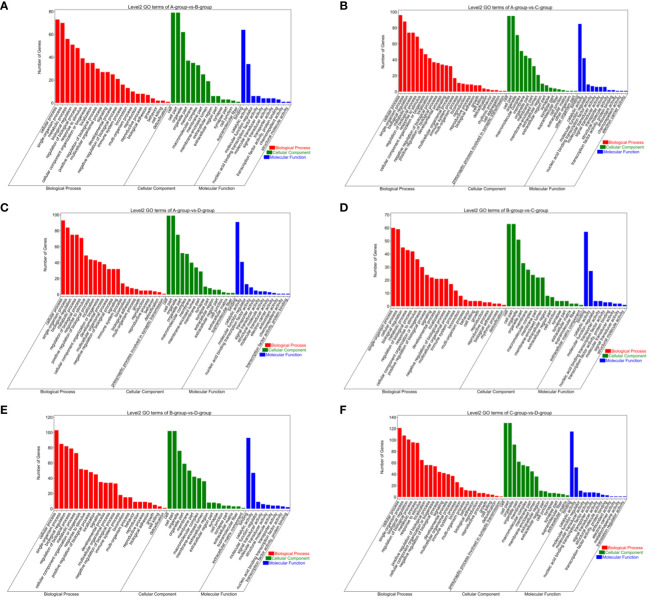
GO enrichment analysis of differentially expressed circRNAs target gene. **(A)** Group A vs Group B; **(B)** Group A vs Group C; **(C)** Group A vs Group D; **(D)** Group B vs Group C; **(E)** Group B vs Group D; **(F)** Group C vs Group D.

The circRNAs are generated through the reverse splicing of exons from protein-coding genes. Annotating the source genes can offer insights into the functions of circRNAs to some extent. In the comparisons between Group A vs Group B, Group A vs Group C, Group A vs Group D, Group B vs Group C, Group B vs Group D, and Group C vs Group D, 83, 105, 103, 65, 109, and 133 differentially expressed circRNA source genes, respectively, were annotated in the GO database. In Group A vs Group B, differentially expressed circRNA source genes are primarily annotated for intracellular (CC), intracellular part (CC), cytoplasmic ribonucleoprotein granule (CC), enzyme binding (MF), high-density lipoprotein particle binding (MF), protein binding (MF), and endothelial cell migration (BP). In Group A vs Group C, differentially expressed circRNA source genes are mainly annotated for macromolecular complex (CC), myosin complex (CC), protein complex (CC), motor activity (MF), transforming growth factor beta-activated receptor activity (MF), protein binding (MF), antigen processing and presentation via MHC class Ib (BP), and antigen processing and presentation of peptide antigen via MHC class Ib (BP). In Group A vs Group D, differentially expressed circRNA source genes are primarily annotated for intracellular (CC), intracellular part (CC), protein complex (CC), binding (MF), lipoprotein particle binding (MF), protein-lipid complex binding (MF), and regulation of catabolic process (BP). In Group B vs Group C, differentially expressed circRNA source genes are mainly annotated for nucleoplasm part (CC), ion binding (MF), binding (MF), kinase binding (MF), antigen processing, and presentation of endogenous peptide antigen via MHC class Ib (BP), and antigen processing and presentation of peptide antigen via MHC class Ib (BP). In Group B vs Group D, differentially expressed circRNA source genes are primarily annotated for intracellular (CC), intracellular part (CC), nuclear inclusion body (CC), clathrin heavy chain binding (MF), ion binding (MF), macromolecular complex binding (MF), and membrane organization (BP). In Group C vs Group D, differentially expressed circRNA source genes are mainly annotated for intracellular (CC), intracellular part (CC), protein complex (CC), ATP binding (MF), adenyl ribonucleotide binding (MF), antigen processing and presentation of endogenous peptide antigen via MHC class Ib (BP), and regulation of metabolic process (BP) (refer to **Figure 13**).

**Figure 14:**
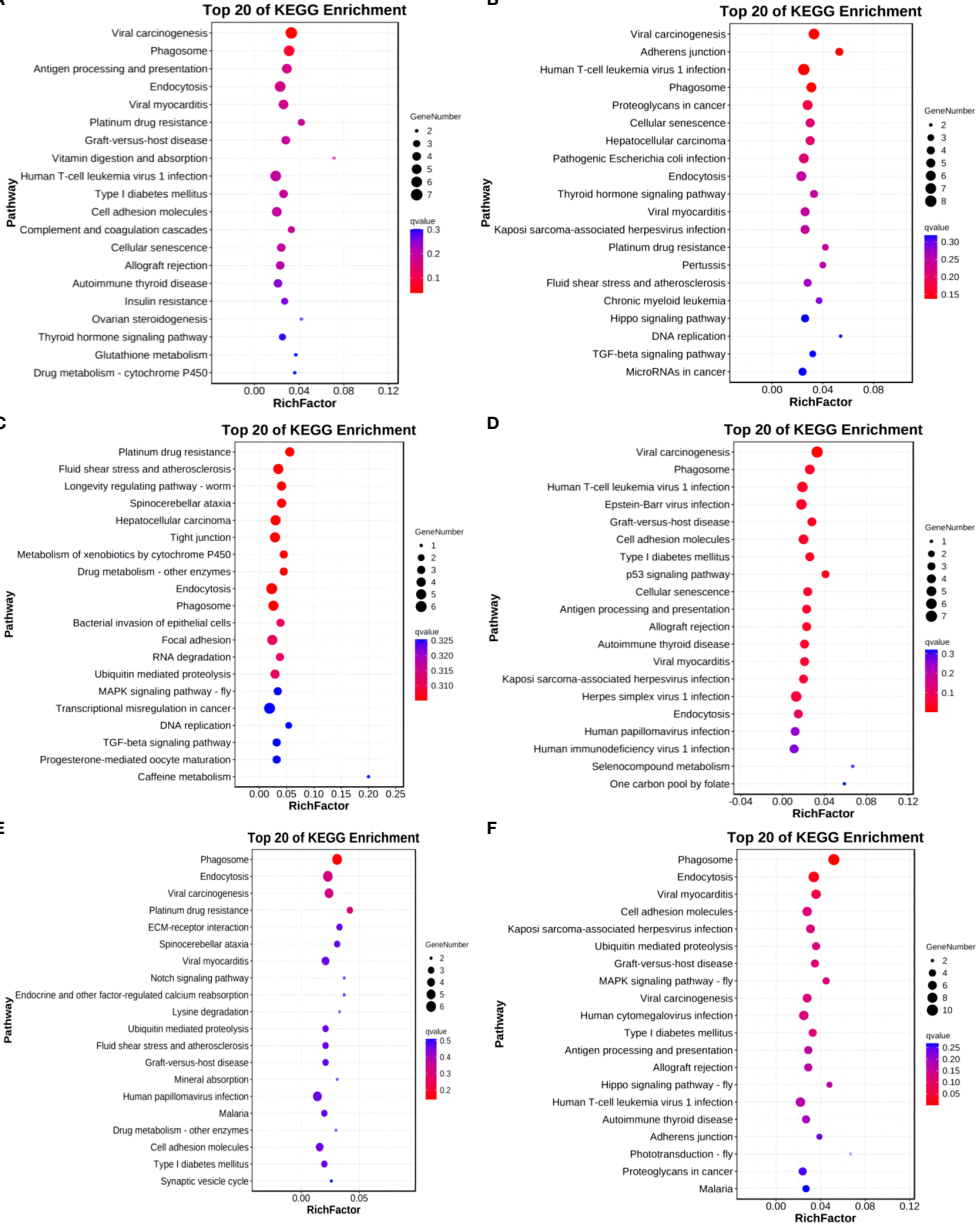
KEGG enrichment analysis of differentially expressed circRNAs target gene. **(A)** Group A vs Group B; **(B)** Group A vs Group C; **(C)** Group A vs Group D; **(D)** Group B vs Group C; **(E)** Group B vs Group D; **(F)** Group C vs Group D.

The KEGG analysis results for differentially expressed circRNA host genes indicate enrichment in 1, 14, and 2 pathways for Group A vs Group B, Group B vs Group C, and Group C vs Group D, respectively (q-value<0.05). No enrichment was observed in the other groups. In Group A vs Group B, the differentially expressed host genes were enriched in the Viral carcinogenesis pathway. In Group B vs Group C, the enriched pathways included Viral carcinogenesis, Phagosome, Human T-cell leukemia virus type 1 infection, Epstein-Barr virus infection, Graft-versus-host disease, and Cellular senescence, among others. In Group C vs Group D, the enriched pathways comprised Phagosome and Endocytosis (refer to **Figure 14**).

### 3.10 Protein-protein interaction

1, 1, 105, 0, 35, and 765 pairs of protein interactions were identified in Group A vs Group B, Group A vs Group C, Group A vs Group D, Group B vs Group D, and Group C vs Group D, respectively. In the protein interaction network of Group A vs Group D, *MCMDC2, KIT, ABL1, ACTA2, RHOJ, RASL11A, PNPLA6, AURKA, MMP2, CALM2* were pinpointed as core nodes. For Group B vs Group D, IHH, KALRN, NCALD, VWCE, ALDH3A2, CORIN, IRS2, TNF, GLIS2, and STK32A emerged as core nodes in the protein interaction network. In Group C vs Group D, MCMDC2, RPS6KA3, MAPK4, PTPRC, BTK, FLT3, RRAD, TNF, KALRN, RAP1B, and others were recognized as core nodes in the protein interaction network (please refer to **Supplementary Figures S1–S3**).

### 3.11 ceRNA network

Finally, a predicted ceRNA network comprised 58 mRNA pairs, 12 lncRNA pairs, and 8 circRNA pairs. For the top 10 connected mRNA/lncRNA/circRNA, a Sankey diagram illustrating their regulatory relationships with miRNAs was constructed. From the diagram, it is apparent that eca-miR-486-3p and miR-486-y exhibit the highest connectivity (please refer to **Supplementary Table S3**, **Figures S4, S5**).

### 3.12 RT-qPCR

**Figure 15:**
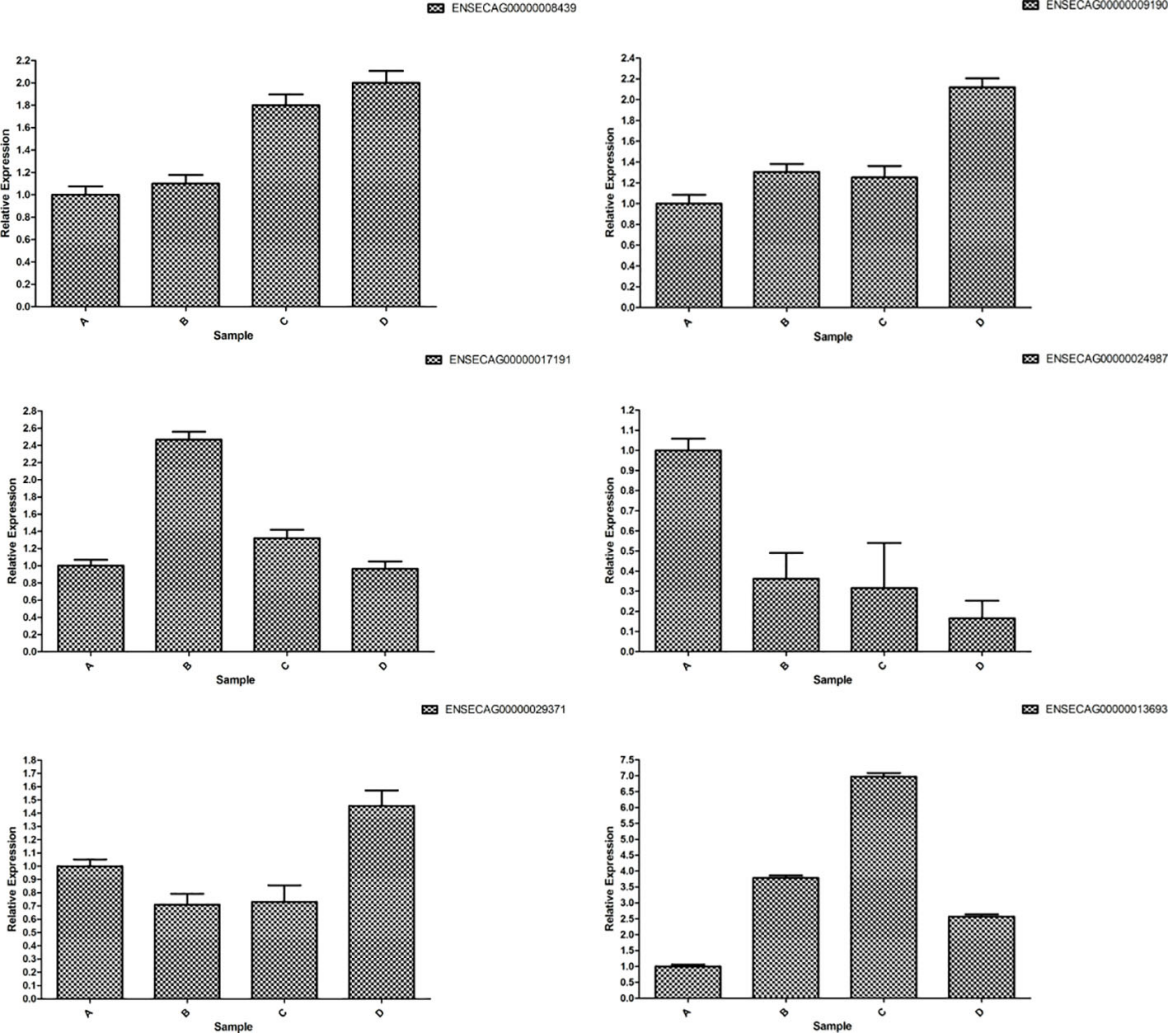
RT-qPCR verification of differently expressed mRNAs.

The consistent trends in the fluorescence quantification results of the six mRNAs with the RNA-Seq data, as depicted in **Figure 15**, affirm the high reliability of the sequencing results.

## 4 Discussion

Ovaries and uterus, as essential reproductive organs, undergo the development and ovulation of dominant follicles on the ovaries, fertilization in the oviduct, and subsequent development in the uterus, leading to parturition under the influence of reproductive hormones. Transcriptomic studies related to ovaries and follicles have been applied to various species, such as humans (30), cows (17, 31, 32), pigs (33, 34), sheep (35), chickens (36), and geese (37). However, research on the ovaries of equine animals, especially horses, is relatively scarce. Horses, as typical seasonal breeders, experience estrus and ovulation influenced by various factors, including age, environment, and nutritional status. Reproductive aging in mares starts around 15 years old, and exceptional mares are essential for breeding high-quality racehorses, exemplified by horses like Dahlia, Allez France, and Oh So Sharp. Therefore, investigating the reproductive mechanism of mares using molecular biology techniques becomes crucial. The Kazakh horse exhibits evident seasonal estrus, occurring 2-3 times per year, with each estrus period lasting for 21 days. According to previous research by our team, mares aged 7-14 years in moderate-to-good body condition and exposed to a temperature range of 18-24°C show optimal estrus conditions. After 15 years of age, reproductive functions begin to noticeably decline, with a distinct pattern of follicular development and higher fertility compared to other age groups. This characteristic makes them an ideal animal model for studying mare follicular development. In-depth research has been conducted globally on the endocrine regulation of ovarian follicular development in equine animals, including mares. However, the specific molecules and cellular processes involved in the regulation of follicular growth and development at different ages in mares remain to be further explored.

In the comparison between groups A, B, C, and D in this study, 22 differentially expressed mRNAs exhibited significant downregulation, while FCER2 (ENSECAG00000009646) demonstrated a notable upregulation. Although existing research on FCER2 predominantly focuses on cancer, it is hypothesized that FCER2 plays a role in the regulation of ovarian aging. The varied expression states of these differentially expressed genes may regulate the proliferation and apoptosis of GCs, leading to follicular atresia or ovarian dysfunction. Among the 23 shared differentially expressed genes, *AUTS2*, *ZFHX2*, *MAST4*, *SLC24A3*, *B3GNT6*, and *PPP1R13L* showed a downregulation, while in Group A vs Group B, Group A vs Group C, and Group B vs Group C, they were upregulated. This suggests that AUTS2 is involved in regulating RNA transcription, splicing, localization, and stability. Follicular development and ovulation are highly complex biological processes, and lncRNA has emerged as a major translational regulatory factor in reproductive processes for humans and animals, such as gonadal development (38) and sex hormone secretion (39). GCs play a highly complex role in oocyte function, involving the regulation of multiple factors. This study suggests that lncRNA plays an important regulatory role in the follicular development and hormonal regulation of mares at different stages before sexual maturity, physical maturity, youth, and old age. By analyzing the lncRNA expression profiles, we identified differentially expressed genes in GCs at different ages in Kazakh horses. It is noteworthy that MSTRG.40650.1 showed significant upregulation, and its expression changed with age, suggesting that MSTRG.40650.1 may be involved in the regulation of aging. In Group C vs Group D, there were the most differentially expressed genes, with 74 upregulated and seven downregulated. MiRNA is a class of endogenous RNAs, approximately 18–24 nucleotides in length, which act by specifically binding to the 3’-UTR region of mammalian mRNAs to inhibit or reduce their translation, thereby post-transcriptionally regulating gene expression (40). Previous studies have shown that miRNAs play crucial roles in cell proliferation, differentiation, growth, migration, and apoptosis (41–44). MiRNAs may inhibit the expression of proteins involved in the proliferation and development of reproductive cells at the post-transcriptional level (45). Eca-miR-34b-3p, miR-12245-y, and miR-21-z show distinct expression patterns when compared under the same conditions, providing substantial evidence for their crucial regulatory roles in the sexual gland development of Kazakh horses. The CircRNA results indicate significant age-related features in the expression profile of GCs in mare ovarian follicle. Notably, novel_circ_001327 showed a downregulation in Group B vs Group C while displaying an upregulation in the other groups. Conversely, novel_circ_009685 exhibited upregulation in the B group compared to the C group but demonstrated downregulation in the other groups. novel_circ_008283 displayed a consistently decreasing trend across all stages. novel_circ_018383 and novel_circ_020541 exhibited an initial increase followed by a rapid decline, reaching their peak expression in the 7-year-old group (Group C) and then rapidly decreasing in mares aged 15 years and above (Group D). This suggests that novel_circ_001327, novel_circ_009685, novel_circ_018383, and novel_circ_020541 may be involved in signaling pathways related to proliferation, differentiation, and reproductive aging. Currently, there is limited research about the involvement of circRNAs, including novel_circ_001327, in ovarian and follicular development, and further studies are needed for validation.

Organisms achieve biological processes through the coordinated action of different genes and their products. The results of the GO enrichment analysis in this study show that differentially expressed genes in ovarian GCs of Kazakh mares at different ages are significantly enriched in biological processes and cellular components. The Mitogen-Activated Protein Kinase (MAPK) cascade is a highly conserved module involved in various cellular functions, including cell proliferation, differentiation, and migration (46). Mammals express at least four distinctly regulated MAPKs: Extracellular Signal-Regulated Kinase (ERK)-1/2, c-Jun N-terminal Kinase (JNK1/2/3), p38 proteins (p38 α/β/γ/δ), and ERK5. These MAPKs are activated by specific MAPKKs: MEK1/2 for ERK1/2, MKK3/6 for p38, MKK4/7 (JNKK1/2) for JNK, and MEK5 for ERK5 (47). However, each MAPKK can be activated by multiple MAPKKKs, increasing the complexity and diversity of MAPK signaling (48). It is speculated that each MAPKKK is responsive to different stimuli. For example, the activation of ERK1/2 by growth factors depends on the MAPKKK c-Raf, while other MAPKKKs may activate ERK1/2 under pro-inflammatory stimuli. The MAPK signaling pathway is one of the important pathways in eukaryotic signal transduction. MAPKs are activated by a series of extracellular stimuli, mediating signals from the cell membrane to the nucleus and regulating various physiological processes such as cell proliferation, differentiation, apoptosis, and death (49, 50). The Hippo signaling pathway is an evolutionarily conserved pathway that controls organ size from flies to humans. In humans and mice, this pathway consists of MST1 and MST2 kinases, their cofactors Salvador, and LATS1 and LATS2 (51, 52). When activated at high cell density, phosphorylated LATS1/2 activates the transcriptional co-activators YAP and TAZ, promoting their cytoplasmic localization, leading to apoptosis and restricting excessive organ growth (53). When the Hippo pathway is inactive at low cell density, YAP/TAZ translocates into the nucleus, binds to the transcription enhancer factor (TEAD/TEF) family of transcription factors, and promotes cell growth and proliferation (54). YAP/TAZ also interact with other transcription factors or signaling molecules, linking processes mediated by the Hippo pathway with other key signaling cascades, such as those mediated by TGF-β and Wnt growth factors (54). The Wnt signaling pathway is essential for the fundamental development of many different species and organs, regulating cell fate determination, progenitor cell proliferation, and controlling asymmetric cell division (55, 56). There are at least three different Wnt pathways: the canonical pathway, the Planar Cell Polarity (PCP) pathway, and the Wnt/Ca^2+^ pathway. In the canonical Wnt pathway, the primary role of Wnt ligands binding to their receptors is to stabilize cytoplasmic β-catenin by inhibiting the degradation complex, thereby stabilizing cytoplasmic β-catenin. Subsequently, β-catenin enters the nucleus freely, interacts with the T-cell factor (TCF) family of transcription factors and associated coactivators, and activates Wnt-regulated genes (57). This indicates that these differentially expressed mRNAs may participate in mare follicular development and the proliferation and apoptosis of GCs through the pathways mentioned above.

Mammalian TLRs are expressed on innate immune cells, such as macrophages and dendritic cells, and respond to membrane components of both Gram-positive and Gram-negative bacteria (58). TLRs recognize pathogens, inducing the production of pro-inflammatory cytokines and upregulating co-stimulatory molecules, thereby rapidly activating the innate immune response (59). The TLR signaling pathway is divided into two groups: the MyD88-dependent pathway, leading to the production of pro-inflammatory cytokines and the rapid activation of NF-Kappa B and MAPK, and the MyD88-independent pathway, inducing INF-β and INF-induced genes and slowly activating NF-Kappa B and MAPK. The MAPK signaling pathway is inhibited by prolactin and is crucial for normal follicular development (59, 60). Cell adhesion molecules are proteins on the cell surface that regulate physiological processes such as cell morphology, migration, proliferation, and differentiation (61). These findings suggest that lncRNAs play a crucial role in regulating protein-coding genes through both cis and trans actions, influencing follicular development in mares during estrus and follicular development. This, in turn, impacts the follicular development of mares at different ages.

Notably, differentially expressed miRNA target genes also exhibit enrichment in the Longevity regulating pathway - multiple species. GCs regulate the development and blockade of follicles through various factors, including gonadotropin receptors, steroid hormones, as well as various growth factors, and cytokines (62–64). Steroid hormones, including estrogen, progestogen, and androgen, directly regulate the maturation of follicles (65). Ras protein, as a GTPase and a molecular switch in signaling pathways, regulates cell proliferation, survival, growth, migration, differentiation, or cytoskeletal dynamics (66). It includes 627 genes, and the Ras signaling pathway is closely linked to 168 pathways, including the MAPK signaling pathway, Calcium signaling pathway, PI3K-Akt signaling pathway, and Regulation of actin cytoskeleton. The FOXO transcription factor family regulates gene expression in cellular physiological events, including apoptosis, cell cycle control, glucose metabolism, oxidative stress resistance, and lifespan (67). Besides PKB, JNK, and AMPK, FOXOs undergo various post-translational modifications, including phosphorylation, acetylation, methylation, and ubiquitination, involving 191,623 genes and 102 pathways, including the MAPK signaling pathway, Cell cycle, and PI3K-Akt signaling pathway. The PI3K-Akt signaling pathway, activated by various stimuli or toxic damage, regulates fundamental cellular functions such as transcription, translation, proliferation, growth, and survival (68). Once activated, Akt controls crucial cellular processes through the phosphorylation of substrates involved in apoptosis, protein synthesis, metabolism, and the cell cycle (69). Gonadotropin-releasing hormone (GnRH) is secreted by the hypothalamus, acts on receptors in the anterior pituitary, and regulates the production and release of gonadotropins, LH, and FSH (70). Downstream signaling of protein kinase C (PKC) leads to the deactivation of epidermal growth factor (EGF) receptors and the activation of MAPKs, including ERK, JNK, and p38 MAPK. Active MAPKs move into the cell nucleus, leading to the activation of transcription factors and the rapid induction of early genes (71). ECM-receptor interaction: The extracellular matrix (ECM) is a complex mixture of structural and functional macromolecules that play a crucial role in tissue and organ morphogenesis, as well as in maintaining the structure and function of cells and tissues (72). Specific interactions between cells and ECM are mediated by transmembrane molecules, primarily integrins, as well as possibly proteoglycans, CD36, or other cell surface-related components (73). These interactions directly or indirectly control cellular activities such as adhesion, migration, differentiation, proliferation, and apoptosis (74). Cellular senescence is an irreversible state of cell growth arrest triggered by various factors such as telomere shortening, oncogene activation, radiation, DNA damage, and oxidative stress (75). Its characteristics include enlarged and flattened morphology, increased activity of senescence-associated β-galactosidase (SA-β-gal), and the secretion of inflammatory cytokines, growth factors, and matrix metalloproteinases, forming part of the senescence-associated secretory phenotype (SASP) (76). Cellular senescence is functionally linked to various biological processes, including aging, tumor suppression, placental biology, and embryonic development. Ovarian Steroidogenesis is critical for normal uterine function, the establishment and maintenance of pregnancy, as well as mammary gland development. The ovarian steroids, 17-β estradiol (E2), and progesterone (P4) play pivotal roles in these processes (77).

This study established networks of protein interaction for differentially expressed genes in GCs of Kazakh horses across various ages, identifying core node genes within each group. Notably, TNF (Tumor Necrosis Factor) emerged as a core node in both Group B vs Group D and Group C vs Group D, exhibiting a significant upregulation in expression across both comparisons. TNF participates in 134 signaling pathways, including the MAPK signaling pathway, NF-kappa B signaling pathway, C-type lectin receptor signaling pathway, and TNF signaling pathway. Previous research indicates that TNF plays a dual role in follicular development across species like pigs and mice, promoting both cell survival and apoptosis. TNF stands out as a crucial cytokine capable of triggering diverse intracellular signaling pathways, encompassing apoptosis, cell survival, inflammation, and immunity. Activated TNF forms homotrimers and binds to its receptors (TNFR1, TNFR2), leading to the trimerization of TNFR1 or TNFR2. TNFR1, expressed in nearly all cells, serves as the primary receptor for TNF (also known as TNF-α). Conversely, TNFR2 is expressed in specific cell types, including CD4 and CD8 T lymphocytes, endothelial cells, microglia, oligodendrocytes, neuronal subtypes, cardiomyocytes, thymocytes, and human mesenchymal stem cells, acting as a receptor for both TNF and LTA (also known as TNF-β). Upon ligand binding, TNFR mediates the recruitment of adaptor proteins (such as TRADD or TRAF2), initiating signal transduction. TNFR1 signaling triggers the activation of numerous genes, predominantly regulated by two distinct pathways: the NF-kB pathway and the MAPK cascade, or cell apoptosis and necroptosis. TNFR2 signaling activates the NF-kappa B pathway, including the PI3K-dependent NF-kappa B pathway and the JNK pathway, promoting cell survival. TNF participates in GC apoptosis through the MAPK signaling pathway, and the apoptosis of GCs is directly linked to follicle development and the decline in ovarian function. The findings of this study suggest that TNF is similarly involved in regulating aging in large mammals beyond mice and humans (78), underscoring its high conservation. Additionally, TNF plays a critical role in multiple signaling pathways, including the mTOR signaling pathway, TGF-beta signaling pathway, and Cytokine-cytokine receptor interaction. Thus, the core nodes regulated by the protein interaction network present potential regulatory factors influencing the follicular development of Kazakh horses at different ages. However, a more in-depth investigation is required to elucidate the specific regulatory mechanisms. Another core node, KALRN, was identified in both Group B vs Group D and Group C vs Group D, exhibiting a significant downregulation in expression across both comparisons. This suggests that KALRN may regulate the proliferation and apoptosis of follicular GCs, thereby influencing follicular development through downregulation.

The ceRNA theory serves as a supplement to the traditional miRNA→RNA paradigm, shedding light on a reverse regulatory mechanism in RNA→miRNA interactions. In this context, ceRNAs competitively engage with the same miRNA recognition elements (MREs), thereby influencing the silencing of target genes triggered by miRNA (79). This theory unveils a novel mechanism of RNA interaction, garnering widespread attention in recent years across various domains, including tumor research, pathological studies, and investigations into growth and development. Currently, research on ceRNA mechanisms is predominantly focused on animals such as cows, pigs, sheep, chickens, and geese, with limited studies on horses. Particularly scarce are investigations into the regulatory network of ceRNA involved in regulating ovarian and follicular development. Through the analysis of mRNA differences in GCs of Kazakh horses at various ages, certain critical candidate genes related to follicular development have become the focal points of our study. Cell growth and differentiation, embryonic development, and the onset and progression of tumors are vital aspects of cellular physiology. The findings of this study reveal that within the ceRNA network, eca-miR-486-3p and miR-486-y have the highest number of nodes. Their target genes participate in multiple signaling pathways associated with reproduction, such as cGMP-PKG signaling, Basal transcription factors, Hippo signaling pathway, Longevity regulating pathway, Ovarian steroidogenesis, Endocrine and other factor-regulated calcium reabsorption, Cortisol synthesis and secretion, and more. Notably, ovarian steroids, 17-β estradiol (E2), and progesterone (P4) play a crucial role in normal uterine function, the establishment and maintenance of pregnancy, and mammary gland development (80). Furthermore, the locally essential actions for normal ovarian physiology rely on the endocrine, paracrine, and autocrine effects of E2, P4, and androgens (81). In most mammals, including humans and mice, ovarian steroidogenesis occurs based on the two-cell/two-gonadotropin theory. This theory elucidates how GCs and follicular cells collaborate to produce ovarian steroids (82, 83). Follicular membrane cells respond to LH signals by increasing the expression of enzymes needed to convert cholesterol into androgens (such as androstenedione and testosterone). GCs respond to FSH signals by increasing the expression of enzymes needed to convert androgens derived from the follicular membrane into estrogens (E2 and estradiol) (84). Cortisol is the primary endogenous glucocorticoid that influences various physiological functions, including lipid and glucose metabolism, metabolic homeostasis, and stress adaptation. Cortisol production is primarily regulated by adrenocorticotropic hormone (ACTH) from the pituitary gland (85). The stimulatory effect of ACTH on cortisol synthesis depends on cAMP-dependent signal transduction but also involves membrane depolarization and cytoplasmic Ca^2+^ increase. Both cAMP and Ca^2+^ induce the expression of StAR, stimulating cholesterol transfer within mitochondria the steroidogenic enzymes (such as CHE, CYP17A1, and CYP11B1) in the cholesterol-to-cortisol pathway (86, 87). While we have constructed a regulatory network related to the mechanisms of action in GCs at different ages and validated the accuracy of the data through RT-qPCR, the intricate mechanisms within the regulatory network still require further in-depth research and discussion.

## 5 Conclusion

This study employed whole transcriptome sequencing to analyze ovarian GCs from a total of 20 Kazakh horses across four different age groups. The results revealed that differentially expressed mRNAs and miRNA target genes were significantly enriched in pathways such as MAPK signaling, Hippo signaling, Wnt signaling, Calcium signaling, Aldosterone synthesis and secretion, Cellular senescence, and NF-kappa B signaling. By constructing protein interaction networks for mRNAs and miRNA target genes, potential regulatory genes associated with reproductive aging were identified. Noteworthy genes for further investigation in the context of horse reproductive aging and regulation include MCMDC2, TNF, and the core regulatory factors eca-miR-486-3p and miR-486-y. Functional validation at the cellular level will be conducted in the future to elucidate the specific mechanisms underlying differential gene expression in our experimental results.

## Data availability statement

The datasets presented in this study can be found in online repositories. The names of the repository/repositories and accession number(s) can be found in the article/**Supplementary Material**.

## Ethics statement

The animal studies were approved by Laboratory Animal Care and Use Ethics Committee at Xinjiang Agricultural University. The studies were conducted in accordance with the local legislation and institutional requirements. Written informed consent was obtained from the owners for the participation of their animals in this study.

## Author contributions

WR: Conceptualization, Methodology, Writing – review & editing, Data curation, Investigation, Validation, Writing – original draft. JW: Investigation, Validation, Writing – original draft, Software. YZ: Visualization, Writing – review & editing. TW: Writing – review & editing, Formal analysis, Validation. JM: Project administration, Resources, Supervision, Writing – review & editing. XY: Writing – review & editing, Conceptualization, Funding acquisition, Methodology, Project administration, Supervision.
