# Supplementary material for: Differential age-related transcriptomic analysis of ovarian granulosa cells in Kazakh horses
Source: Front Endocrinol (Lausanne). 2024 Jan 30;15:1346260. doi: 10.3389/fendo.2024.1346260 (PMC10863452; doi:10.3389/fendo.2024.1346260)

Differentially expressed gene protein interaction network and core gene selection(C-Group-VS-D-Group).

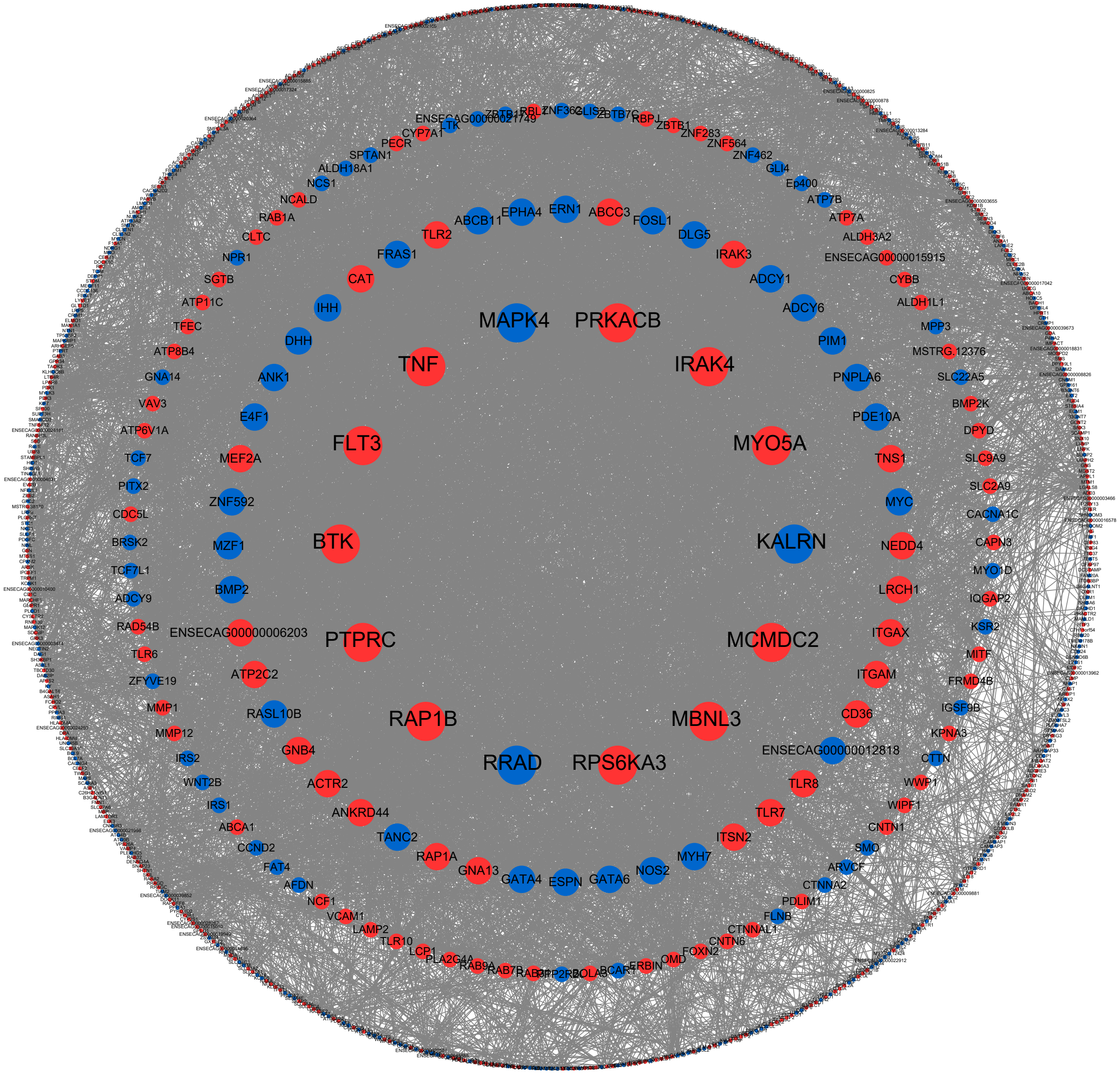

Supplement: Supplementary file 1 [file DataSheet_1.zip › Supplementary Material 20240122/Figure S3 PPI C-group-vs-D-group.pdf]
