# Supplementary material for: Differential age-related transcriptomic analysis of ovarian granulosa cells in Kazakh horses
Source: Front Endocrinol (Lausanne). 2024 Jan 30;15:1346260. doi: 10.3389/fendo.2024.1346260 (PMC10863452; doi:10.3389/fendo.2024.1346260)
Supplement: Supplementary file 1 [file DataSheet_1.zip › Supplementary Material 20240122/Table S1 Information on differentially expressed mRNA primers.docx]

**Table S1:** **Information on differentially expressed mRNA primers**

| Symbol |  | Primer 5'-3' | Tm (℃) | Product (bp) | Description |
| --- | --- | --- | --- | --- | --- |
| *FTL* | F | CGAGTTGGCGGAGGAGA | 56.9 | 342 | ferritin light chain [Source:NCBI gene;Acc:100051593] |
|  | R | AAGAGATACTCGCCCAGCC | 56.7 |  |  |
| *SPP1* | F | CGTGGCTTCTGATTGGGACA | 61.5 | 242 | secreted phosphoprotein 1 [Source:VGNC Symbol;Acc:VGNC:23533] |
|  | R | CTTCGCTCTTACGGTCTCGG | 60.1 |  |  |
| *TMSB4X* | F | ACCTCGCTCCGCTTCCTCT | 61.2 | 155 | thymosin beta 4 X-linked [Source:NCBI gene;Acc:100034015] |
|  | R | ATTCGCCTGCTTGCTTCTCC | 61.9 |  |  |
| *HMGB1* | F | CGGGAGGAGCACAAGAAGAA | 60.1 | 265 | high mobility group box 1 [Source:NCBI gene;Acc:100033873] |
|  | R | TGGGGCGATACTCAGAACAA | 58.8 |  |  |
| *COL1A1* | F | AGCAAGAACCCCAAGGACAA | 59.5 | 295 | collagen type I alpha 1 chain [Source:VGNC Symbol;Acc:VGNC:16730] |
|  | R | CGTAGGTGACGCTGTAGGTG | 56.4 |  |  |
| *FAM234A* | F | AACCGAGCGAAGAAGGAAGA | 59.1 | 327 | family with sequence similarity 234 member A [Source:NCBI gene;Acc:100066481] |
|  | R | GCAGGGAGAGGAAAAGCC | 56.9 |  |  |
| *GAPDH* | F | TGGTGAAGGTCGGAGTAAACGG | 63.8 | 372 | glyceraldehyde-3-phosphate dehydrogenase [Source:NCBI gene;Acc:100033897] |
|  | R | CAGCAGAAGGAGCAGAGATGA | 57.9 |  |  |
| *β-Actin* | F | TCTGCTATGTCGCCCTGGA | 59.6 | 115 | actin beta [Source:NCBI gene;Acc:100033878] |
|  | R | CGCTCGTTGCCGATGGT | 60.6 |  |  |
