# Supplementary material for: Differential age-related transcriptomic analysis of ovarian granulosa cells in Kazakh horses
Source: Front Endocrinol (Lausanne). 2024 Jan 30;15:1346260. doi: 10.3389/fendo.2024.1346260 (PMC10863452; doi:10.3389/fendo.2024.1346260)
Supplement: Supplementary file 1 [file DataSheet_1.zip › Supplementary Material 20240122/Table S3 mRNA, lncRNA, circRNA, and miRNA targeting relationship statistics.docx]

Table S3: Statistics of final ceRNA pair

| Type | ncRNA_num | mRNA_num | Pairs_num | miRNA_num |
| --- | --- | --- | --- | --- |
| lncRNA | 12 | 48 | 58 | 23 |
| circRNA | 8 | 15 | 16 | 10 |
| total | 20 | 58 | 74 | 30 |
