# Supplementary figures and images for: Differential age-related transcriptomic analysis of ovarian granulosa cells in Kazakh horses
Source: Front Endocrinol (Lausanne). 2024 Jan 30;15:1346260. doi: 10.3389/fendo.2024.1346260 (PMC10863452; doi:10.3389/fendo.2024.1346260)

mRNA degree distribution

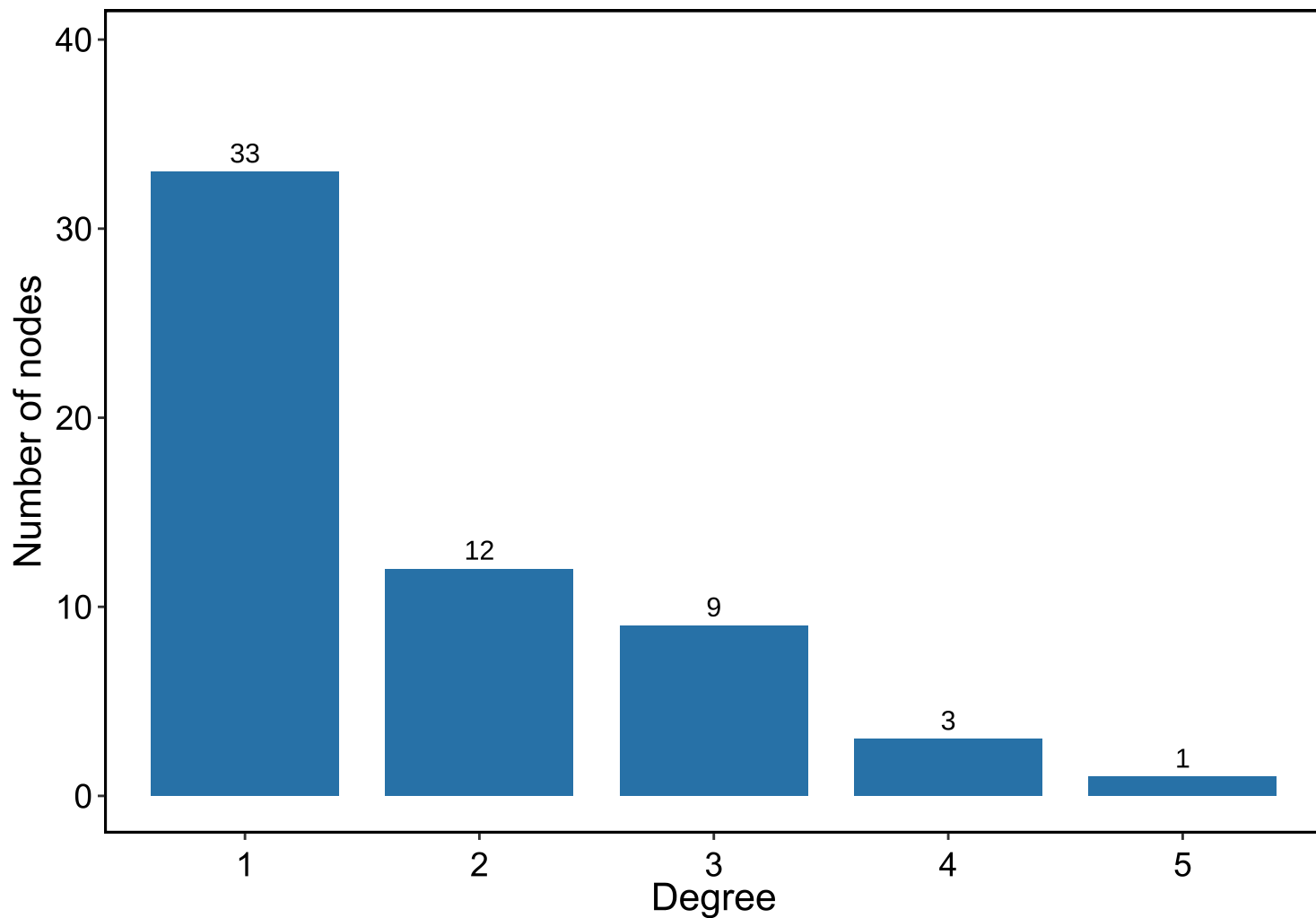

IncRNA degree distribution

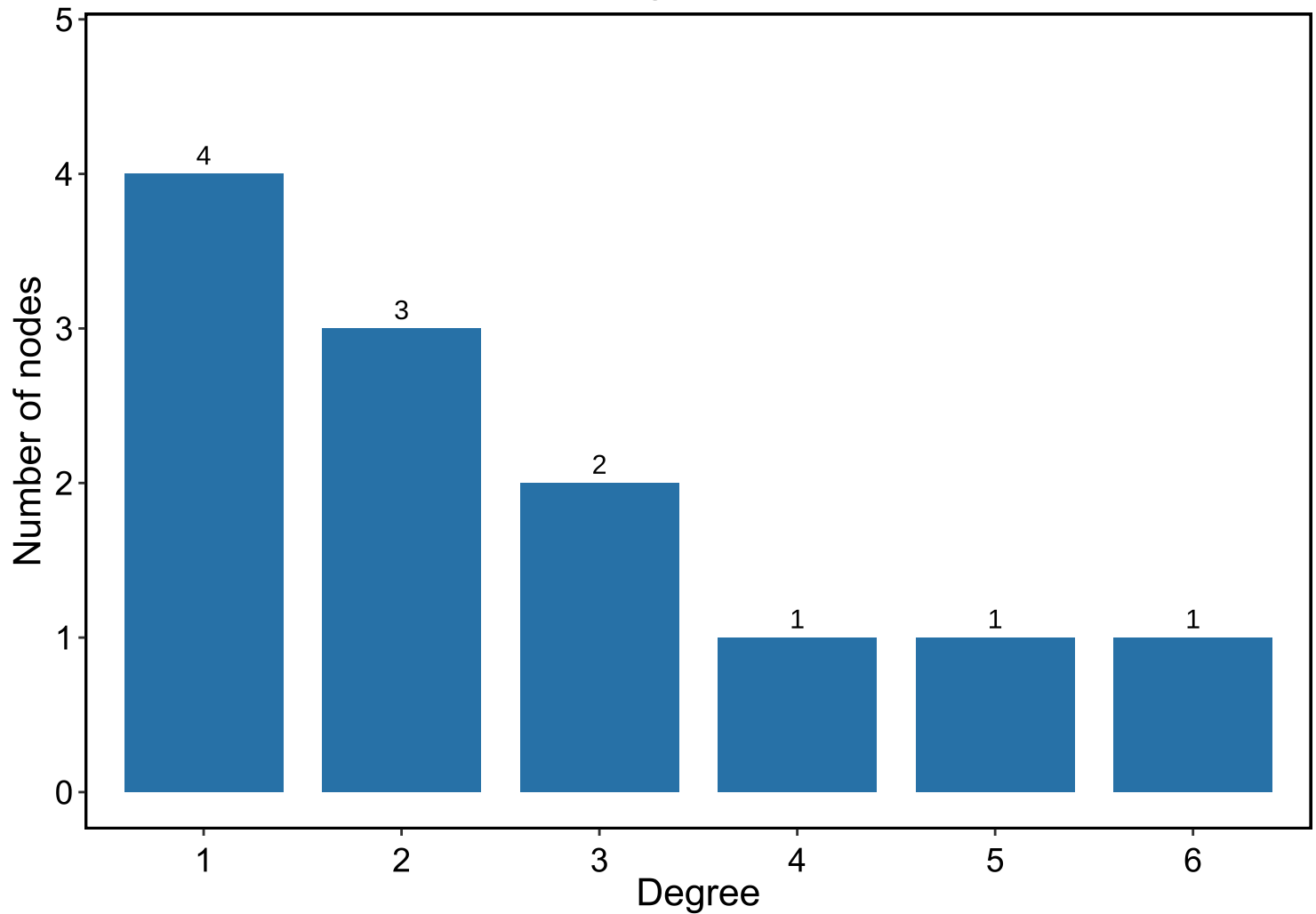

circRNA degree distribution

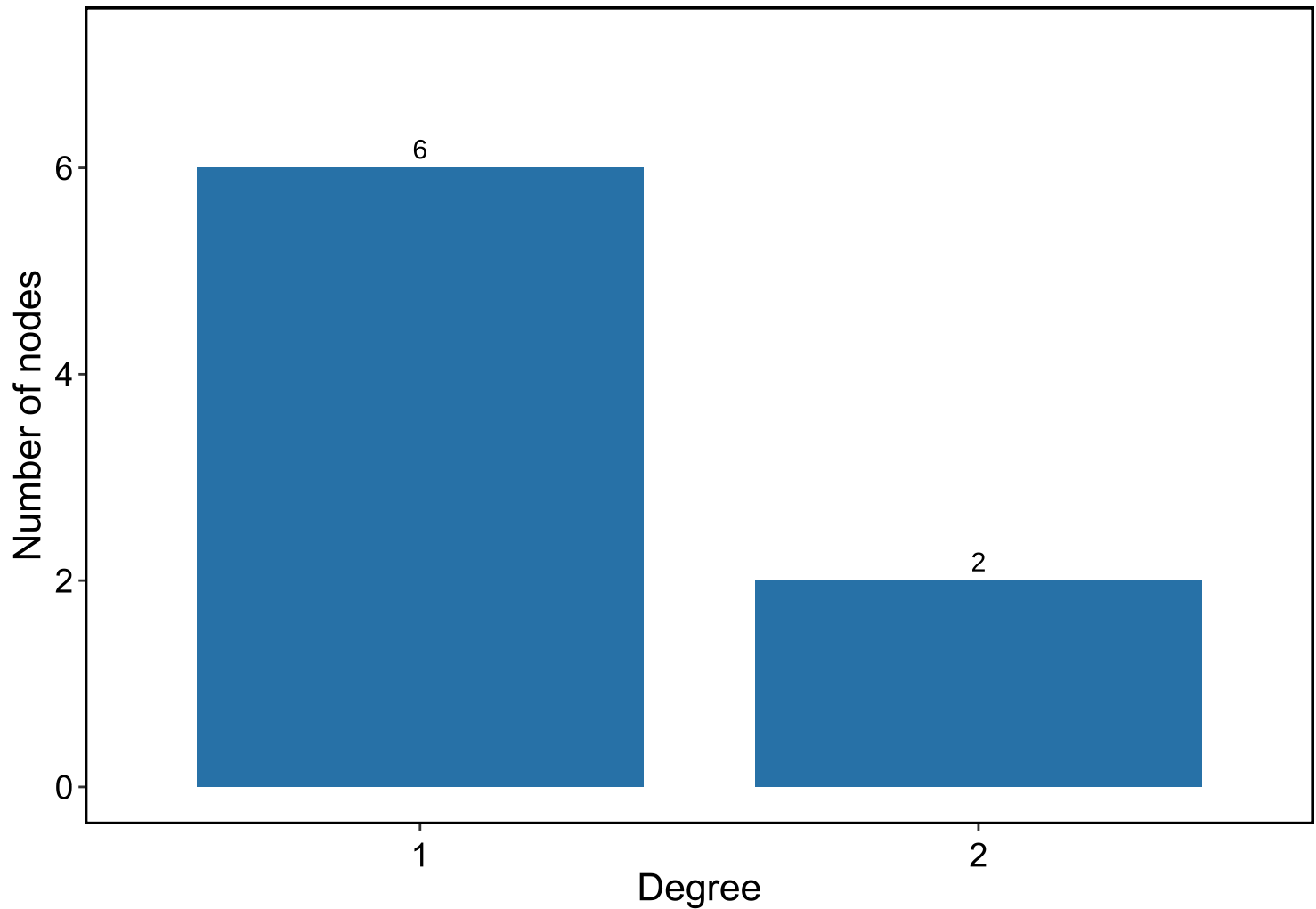

Supplement: Supplementary file 1 [file DataSheet_1.zip › Supplementary Material 20240122/Figure S4 Information on differentially expressed miRNA primers.pdf]

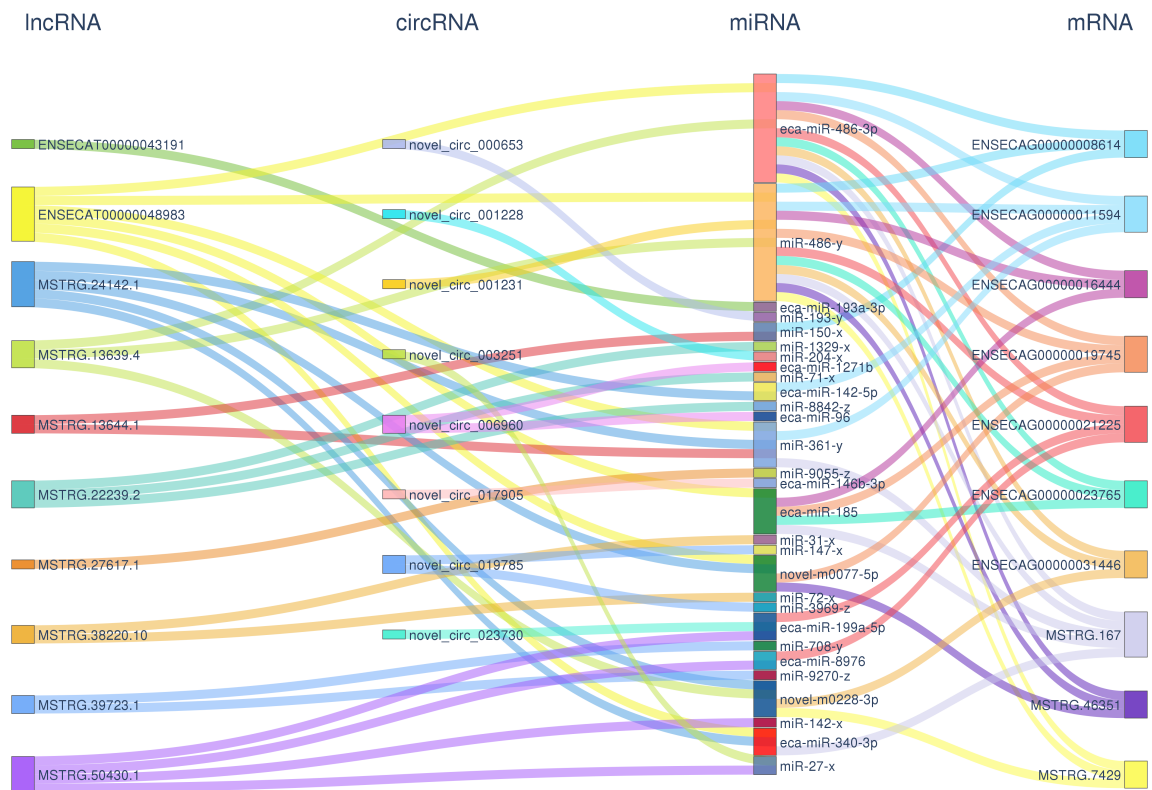

Supplement: Supplementary file 1 [file DataSheet_1.zip › Supplementary Material 20240122/Figure S5 ceRNA network diagram.pdf]
